# Projecting years in good health between age 50–69 by education in the Netherlands until 2030 using several health indicators - an application in the context of a changing pension age

**DOI:** 10.1186/s12889-022-13223-8

**Published:** 2022-04-29

**Authors:** Jose R. Rubio Valverde, Johan P. Mackenbach, Anja M. B. De Waegenaere, Bertrand Melenberg, Pintao Lyu, Wilma J. Nusselder

**Affiliations:** 1 Department of Public Health, Erasmus MC, Rotterdam, the Netherlands; 2 School of Economics and Management, Tilburg University, Tilburg, the Netherlands

**Keywords:** Ill-health, Retirement, Socioeconomic position

## Abstract

**Objective:**

We investigate whether there are changes over time in years in good health people can expect to live above (surplus) or below (deficit) the pension age, by level of attained education, for the past (2006), present (2018) and future (2030) in the Netherlands.

**Methods:**

We used regression analysis to estimate linear trends in prevalence of four health indicators: self-assessed health (SAH), the Organization for Economic Co-operation and Development (OECD) functional limitation indicator, the OECD indicator without hearing and seeing, and the activities-of-daily-living (ADL) disability indicator, for individuals between 50 and 69 years of age, by age category, gender and education using the Dutch National Health Survey (1989–2018). We combined these prevalence estimates with past and projected mortality data to obtain estimates of years lived in good health. We calculated how many years individuals are expected to live in good health above (surplus) or below (deficit) the pension age for the three points in time. The pension ages used were 65 years for 2006, 66 years for 2018 and 67.25 years for 2030.

**Results:**

Both for low educated men and women, our analyses show an increasing deficit of years in good health relative to the pension age for most outcomes, particularly for the SAH and OECD indicator. For high educated we find a decreasing surplus of years lived in good health for all indicators with the exception of SAH. For women, absolute inequalities in the deficit or surplus of years in good health between low and high educated appear to be increasing over time.

**Conclusions:**

Socio-economic inequalities in trends of mortality and the prevalence of ill-health, combined with increasing statutory pension age, impact the low educated more adversely than the high educated. Policies are needed to mitigate the increasing deficit of years in good health relative to the pension age, particularly among the low educated.

**Supplementary Information:**

The online version contains supplementary material available at 10.1186/s12889-022-13223-8.

## Introduction

The demographic processes of increasing longevity [1, 2], with a reduction in the working-age population put pressure on already strained pension systems in Europe. This has led governments to implement policies that raise the statutory pension age and reduce incentives to retire early. Most pension reforms automatically linked future pensions to projected changes in life expectancy [3]. These policies do not account for the socio-economic stratification of society, where individuals of lower strata tend to live not only shorter lives [4], but also less years in good health [5, 6], with the gap being generally larger for life expectancy in good health.

Poor physical and mental health are important determinants of premature labor market exit. Poor self-reported health [7–12], chronic conditions [10, 12], functional limitations [7], disability [13] and poor mental health [14] are linked with an increased risk of exiting the labor market in European countries. Inequalities across many health indicators are prevalent and persistent between education levels [15]. Low educated individuals experience worse physical [16, 17] and mental health [18, 19] than high educated individuals and poor health is associated with higher risks to exit the labor force prematurely due to disability pension and unemployment [20, 21].

The need to look beyond trends in life expectancy of the national population to assess the feasibility of changes in the statutory pension age is increasingly acknowledged. Health expectancy indicators for different socioeconomic groups are used for this purpose and they show large, persistent and in most countries increasing inequalities [22]. This raises concerns that groups in the population will not be entitled to a state pension after they reach the end of their healthy life because they have not yet reached the revised pension age [23]. However, a quantification of the deficit in years in good health prior to the increased pension age is generally lacking. Studies on trends in life expectancy in good health for different socioeconomic groups provide some indication of the unequal impact of the increasing pension age, but may mask relevant developments for the ages around the pension age, because changes in this indicator also reflect trends in mortality and health of persons in their seventies and older. The study of Majer et al. [5] examined socioeconomic inequalities in health expectancy between age 50 and 65 years in 10 Western-European countries to avoid this, but used data for the period 1995–2001, prior to the increase in pension age in most countries.

The Netherlands is an example of a country that has increased and is further increasing the statutory pension age. The statutory pension age was fixed at 65 years until 2013. Following this, it increases stepwise to 67 years in 2024. After this year, it was set to increase at a rate of 8 months per 1-year increase in projected life expectancy at age 65 [24]. A recent Dutch study [25] found an increase in the prevalence of individuals with health problems at the increased pension age. However, this study did not include different socioeconomic groups, nor information about health prior to retirement, which is needed to assess how much earlier the healthy life ends than the pension age.

We present the expected deficit of the number of years in good health before reaching the pension age or the surplus of the number of years in good health after reaching the pension age by education level, using four health indicators that are relevant for labor market participation and are associated with premature exit from the labor market. Considering the changes to the pension age in the Netherlands, we select three points in time with different statutory pension ages: 1) the period when the statutory age still was 65 (2006), 2) a period close to the present (2018 with 66 years), and 3) a period in the future showing what is expected if the observed trends continue (2030 with 67.25 years). Our study provides insights into changes in inequalities in years in good health and how these changes interact with the increasing pension age in the Netherlands.

## Data and methods

### Data

#### Health indicators by education

We used the 1989–2018 cross-sectional waves of the Dutch Health Interview Survey conducted by Statistics Netherlands [26, 27] to obtain data on four health indicators by educational group (See Additional file 1: Appendix Table 1). This is a representative survey among persons living in private households with a response rate of about 60–65%. Additional file 1: Appendix Table 2 contains information on sample sizes.

We based our classification on the survey question about the highest level of completed education. We combined categories of the highest level of education attained to form three levels of education: lowest level, medium level and highest level, corresponding to ISCED categories 0–2, 3–4 and 5–6 respectively. For reasons of brevity, throughout the remainder of the text, we use the terms ‘low’, ‘mid’ and ‘high’ educated. We used education because it is generally completed in early adulthood, it is a stable measure of socio-economic status and is less affected by reverse causation [28].

We included four health indicators in our analyses which have been shown to impact labor market outcomes [7–12].

##### Self-assessed health (SAH)

The survey contained the question “In general, how do you consider your health status”. We categorized it into reporting at least good health (very good and good) and less than good health (fair, bad, very bad).

##### Organization for Economic Cooperation and Development (OECD) functional limitation indicator

The survey includes a set of questions aimed to assess the presence of several functional limitations. These include limitations in hearing, seeing and mobility [29]. Individuals are classified as having OECD functional limitations if they report “Yes, with great difficulty” and “No, I cannot” for least one limitation.

##### OECD without hearing and seeing

We also used the OECD functional limitations excluding the hearing and seeing items because the change over time for these items may depend strongly on innovations regarding hearing and seeing devices and in the scientific literature these items are generally not included.

##### Activities of daily living (ADLs)

The survey includes information on ADL disability for individuals over the age of 55. These include limitations in eating and drinking, dressing, moving around, washing themselves and in going up and down stairs. Individuals are classified as having ADL disability if they report “Yes, with great difficulty” or “Only with help from others” for at least one ADL.

We did not include chronic conditions as health indicator, since chronic conditions may not have consequences on labor market outcomes if successfully treated, e.g. with medications or surgery. Mental health indicators could not be included because they were not part of the Dutch Health Survey for the period we studied, however some of the indicators in our study, including SAH [30] and ADL [31] capture in part mental health. OECD without hearing and seeing was included as robustness check to assess to what extend the trends in the OECD limitations were driven by changes in hearing and seeing.

### Mortality by education

The mortality rates by gender, age group (50–54; 55–59; 60–64; 65–69) and education (low, medium and high) for the Netherlands for the years 2006, 2016 and 2030 were obtained from a recent paper on projections of life expectancy by education for the Netherlands (Nusselder et al.: Future trends of life expectancy by education in the Netherlands, Submitted). This projection used the same classification of education as the survey data. Data on deaths and person years for the period 2006–2018 were based on individual data linkage of different data sources in the secure environment of Statistics Netherlands. Data on the educational attainment was based on the Educational Attainment File constructed by Statistics Netherlands by combining information on education levels from several registers. There was no information on educational attainment for every citizen in the population, therefore weights were used in combination with a calibration procedure developed by Statistics Netherlands [32].

The projections of future mortality were based on a three-layered Lee and Li approach [33]. This approach used additional data from five North-Western European countries. The upper layer models a common trend (not by education) for the Netherlands and 5 other North-Western European countries, the second layer models the deviation of education-specific mortality from the common trend, and the third layer the deviation of Dutch education-specific mortality from international education-specific mortality of the selected countries. This approach was used to 1) create a broader empirical basis for the identification of the most likely long-term trend, and 2) to combine longer time series on national mortality data with shorter series on mortality by education at the European level and similarly, to combine longer time series on mortality by education at the European level with shorter time series by education for the Netherlands. Including mortality data from other countries to create a broader empirical basis is also used in national projections [34]. Deviations of mortality in the Dutch education groups from the international education groups were very small and behaved like random noise. More details on the mortality projections including the selection of the countries are given in Additional file 1: Appendix 3 and in the underlying paper (Nusselder et al.: Future trends of life expectancy by education in the Netherlands, Submitted).

## Methods

### Health indicator prevalence

We estimated logistic regression models with the dichotomous health indicators as dependent variable, and age (50–54; 55–59; 60–64; 65–69), education (low, medium, high), year of the survey (as a continuous variable), and an interaction term between education and year as independent variables.

Based on these logistic regression models we obtained estimates of the prevalence of poor health between 1989 and 2030 by education, of the absolute and relative inequalities in prevalence, and of time trends in the prevalence by education for each health indicator. We used the *margins* command in STATA to calculate past and future prevalence of poor health. Margins involves predicting the probability of poor health for each observation in the sample (using the estimated coefficients and the respective covariate values) and then averaging over all the individuals in the sample [35]*.* We used the *adjrr* command in STATA to calculate risk differences and risks ratios based on the predicted prevalence by education based on the margins command. Risk differences measure the absolute difference in prevalence between low and high educated (prevalence low-prevalence high), risk ratios the relative differences (prevalence low/prevalence high). Finally, we used the *margins (dydx)* command (average marginal effects) to calculate the average change over 1 year in the prevalence of each of the health indicators. This corresponds to the expected difference in the prevalence of the health outcome associated with a unit increase in time, adjusted to the sample distributions of the variables included in the models. All models were stratified by gender.

For robustness checks we ran two sets of additional models and used likelihood-ratio (LR) tests and Akaike’s information criterion to compare the fit with the main models. The first used cubic splines for calendar year to check for non-linear trends. The LR tests indicated a better fit for models with cubic year splines for men for the OECD indicator without hearing and seeing and for women for both OECD indicators. The Akaike’s Information Criterion, however showed that the preference for the cubic spline is only modest relative to our main models. The second set of additional models included a three-way interaction term between age category, education and year. The LR test and Akaike’s Information Criterion showed that adding the interaction improved the fit for men for SAH and for women for SAH and both OECD indicators. The results for the prevalence trends by education were similar when including the interaction. Since these alternative model specifications did not consistently and only modestly improved the model fit, and because comparability between the health indicators is important in our study, we focus on the outcomes of the main models. Details on the robustness checks are given in Additional file 1: Appendix 4.

We estimated the observed age-standardized prevalence of each health indicator by gender, education and year using the 2013 European standard population [36] to compare with the predicted prevalences based on the logistic regression model.

All analyses used survey weights and robust standard errors and were conducted using STATA v15.

### Years in good health

We used the Sullivan method [37] to calculate years lived in good health between ages 50 and 69 for each of the health indicators by level of education and gender, using the age-specific past and projected mortality rates and prevalence of poor health. The Sullivan method uses the prevalence of poor health in each age group to divide the number of person years into years in good and poor health. We used period life tables for the estimation of life expectancy and years in good health.

### Surplus and deficit of years in good health relative to the pension age

We compared for the three selected years for each health indicator the years in good health between ages 50 and 69 and the years between age 50 and the statutory pension age for that specific year (using: Years in good health between ages 50 and 69 at year t – (pension age at year t-50)). If this difference is negative, there is a deficit of years in good health, and if it is positive, a surplus. In 2006 the statutory pension age was 65 years, in 2018 66 years and in 2030 it will be 67 years and 3 months (based on current regulations and the current projection of Statistics Netherlands [38]). We present the deficit/surplus of years in good health by education and gender.

We also estimated the difference between high and low educated in deficit/surplus years, providing a measure of absolute inequality of deficit/surplus. In addition, to assess the contribution of changes in mortality and changes in health to inequalities in deficit/surplus, we estimated these inequalities assuming constant poor health and mortality, both separately and simultaneously.

## Results

### Health Indicator prevalence

Table 1 shows the risk ratios and risk differences summarizing the results of the logistic regression analyses. The top row shows an increase in prevalence as age increases for all health indicators for women, but for men the prevalence of the age group 60–64 is often higher than that of age group 65–69.

**Table 1:** Adjusted risk ratios and risk difference and average change over 1 year for health indicators using the Dutch Health Survey (1989–2018), stratified by gender

**Men**									
		**Less than good self-reported health**		**OECD disability indicator (≥ 1)**		**OECD without hearing and seeing(≥ 1)**		**Activities of Daily Living -ADL (≥ 1)**	
		*Risk ratio*	*Risk Difference*	*Risk ratio*	*Risk Difference*	*Risk ratio*	*Risk Difference*	*Risk ratio*	*Risk Difference*
Age category ^a^	50–54	1.00	0.00	1.00	0.00	1.00	0.00	0	0
		(ref)	(ref = 25.34)	(ref)	(ref = 13.42)	(ref)	(ref = 6.35)		
	55–59	**1.16**	**4.15**	1.07	0.95	**1.21**	**1.34**	1.00	0.00
		(0.00)	(0.00)	(0.10)	(0.10)	(0.00)	(0.00)	(ref)	(ref = 4.74)
	60–64	**1.24**	**6.03**	1.09	1.19	**1.30**	**1.88**	**1.16**	**0.77**
		(0.00)	(0.00)	(0.13)	(0.13)	(0.00)	(0.00)	(0.04)	(0.04)
	65–69	**1.13**	**3.30**	1.03	0.45	**1.17**	**1.10**	1.11	0.52
		(0.00)	(0.00)	(0.93)	(0.93)	(0.01)	(0.01)	(0.16)	(0.16)
Average Educational inequalities ^a^	High	1.00	0.00	1.00	0.00	1.00	0.00	1.00	0.00
		(ref)	(ref = 17.36)	(ref)	(ref = 7.84)	(ref)	(ref = 2.78)	(ref)	(ref = 1.91)
	Medium	**1.50**	**8.71**	**1.66**	**5.18**	**2.26**	**3.55**	**2.45**	**2.79**
		(0.00)	(0.00)	(0.00)	(0.00)	(0.00)	(0.00)	(0.00)	(0.00)
	Low	**2.21**	**21.12**	**2.78**	**13.94**	**4.16**	**8.83**	**4.01**	**5.77**
		(0.00)	(0.00)	(0.00)	(0.00)	(0.00)	(0.00)	(0.00)	(0.00)
Average absolute change in prevalence over 1 year by education level ^b^	High	0.05		**−0.10**		0.00		0.02	
		(0.32)		(0.04)		(0.84)		(0.39)	
	Medium	−0.10		**−0.16**		−0.02		0.05	
		(0.06)		(0.00)		(0.52)		(0.08)	
	Low	0.04		**−0.14**		0.04		**0.11**	
		(0.93)		(0.010)		(0.33)		(0.01)	
n		34,548		28,508		28,699		22,804	
**Women**									
		**Less than good self-reported health (SAH)**		**OECD disability indicator**		**OECD without hearing and seeing**		**Activities of Daily Living (ADL)**	
		*Risk ratio*	*Risk Difference*	*Risk ratio*	*Risk Difference*	*Risk ratio*	*Risk Difference*	*Risk ratio*	*Risk Difference*
Age category ^a^	50–54	1.00	0.00	1.00	0.00	1.00	0.00	0	0
		(ref)	(ref = 29.35)	(ref)	(ref = 19.22)	(ref)	(ref = 12.12)		
	55–59	**1.06**	**1.67**	1.00	0.00	1.06	0.79	1.00	0.00
		(0.02)	(0.02)	(0.97)	(0.97)	(0.19)	(0.18)	(ref)	(ref = 6.35)
	60–64	**1.17**	**5.00**	1.05	0.98	**1.22**	**2.77**	**1.30**	**1.92**
		(0.02)	(0.00)	(0.17)	(0.17)	(0.00)	(0.00)	(0.00)	(0.00)
	65–69	**1.18**	**5.14**	**1.16**	**3.24**	**1.42**	**5.14**	**1.50**	**3.16**
		(0.00)	(0.00)	(0.00)	(0.00)	(0.00)	(0.00)	(0.00)	(0.00)
Average Educational inequalities ^a^	High	1.00	0.00	1.00	0.00	1.00	0.00	1.00	0.00
		(ref)	(ref = 21.32)	(ref)	(ref = 10.74)	(ref)	(ref = 6.69)	(ref)	(ref = 4.16)
	Medium	**1.34**	**7.40**	**1.54**	**5.82**	**1.80**	**5.16**	**1.56**	**2.33**
		(0.00)	(0.00)	(0.00)	(0.00)	(0.00)	(0.00)	(0.00)	(0.00)
	Low	**1.75**	**16.03**	**2.36**	**14.62**	**2.67**	**11.17**	**2.32**	**5.53**
		(0.00)	(0.00)	(0.00)	(0.00)	(0.00)	(0.00)	(0.00)	(0.00)
Average absolute change in prevalence over 1 year by education level ^b^	High	−0.06		**−0.14**		−0.06		−0.05	
		(0.44)		(0.02)		(0.29)		(0.27)	
	Medium	0.08		**−0.11**		−0.04		0.04	
		(0.22)		(0.03)		(0.43)		(0.41)	
	Low	**0.13**		−0.01		**0.09**		**0.10**	
		(0.00)		(0.77)		(0.05)		(0.01)	
n		35,307		29,079		29,392		22,793	

^a^Estimates are derived from logistic regression models including age category (50–54;..;65–69), education level (low, medium, high), year of the survey, and interaction term between education and year. Adjusted risk differences and ratios are derived using the post-estimation command *adjrr* in STATA. Reference prevalence corresponds to the model predicted prevalence for the average of all years in the sample. *P*-values in parenthesis

^b^ Estimates are derived from the post-estimation command *margins, dydx* in STATA, corresponding to the average marginal (partial) effects, meaning that the effects are calculated for each observation in the sample and then averaged

The middle row of Table 1 shows that the prevalence for all health indicators was higher for the low educated when compared to the high educated. The highest average absolute inequalities occur for less than good SAH, with 21.1% prevalence difference between the low and high educated for men and 16.0% prevalence difference for women. The highest relative inequalities are observed for the OECD indicator without the hearing and seeing items for men, with a prevalence ratio of 4.2 between low and high educated. For women, the highest relative inequalities occur for the same indicator, with a prevalence ratio of 2.7.

The last row of Table 1 shows the average change in prevalence over 1 year for each of the health indicators by education, controlling for age. For men, there is a significant increase over time for low educated for the ADL prevalence of 0.11 percentage points per year. There is a significant decrease in the OECD prevalence for all education levels. The trends for the less-than-good SAH indicator are not statistically significant. For low educated women, there is a significant increase in the prevalence of less-than-good SAH, the OECD indicator without hearing and seeing and the ADL indicator. High educated women experienced a decrease for all indicators but only significant for the OECD indicator.

**Fig. 1:**
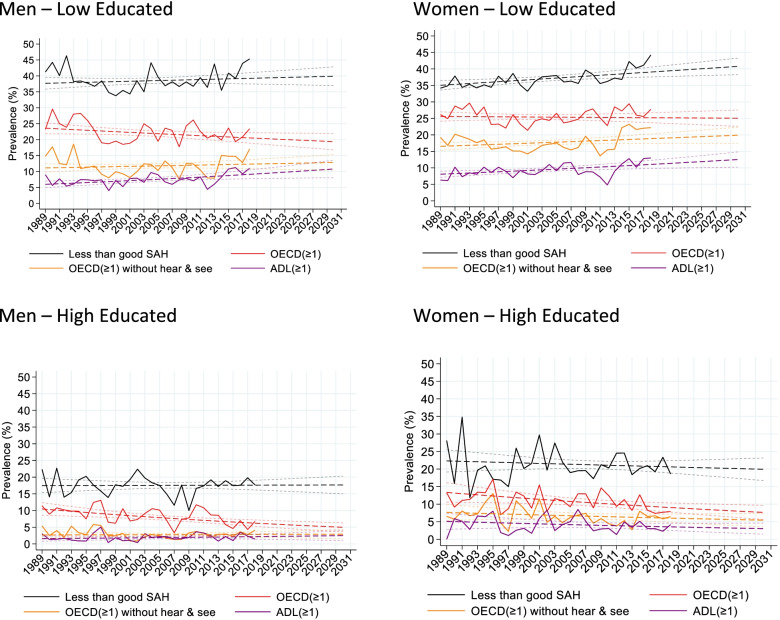
Age-standardized prevalence of health indicators for the Netherlands from the Health Interview survey for individuals aged 50–69 by year, gender, education

Figure 1 presents the age-standardized prevalence of the four health indicators over time by education and gender, based on the observed prevalence (1989–2018) and the extrapolated prevalence (2019–2030) by age (for tables see Additional file 1: Appendix 5). This overall picture is in line with the regression results. For both genders, low educated have higher age-standardized prevalence of poor health for all indicators than high educated. Comparing the figures for low and high educated, shows that particularly for women the gap between low and high educated widens over time.

### Years in good health

**Fig. 2:**
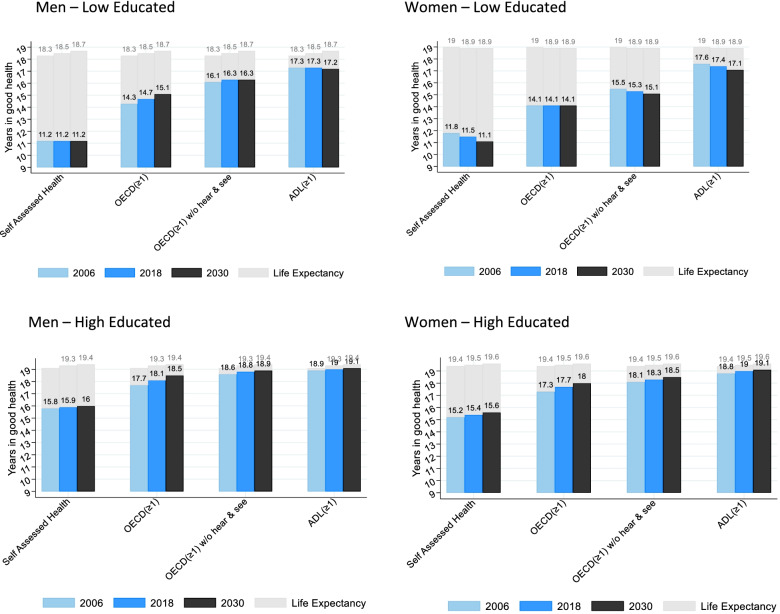
Years in good health for different health indicators and life expectancy between ages 50–69 by year, gender, education level

Figure 2 shows the expected years in good health for the four health indicators for 2006, 2018 and 2030 by education and gender based on the age-specific prevalences of poor health and mortality rates for past and future years (for tables see Additional file 1: Appendix 6).

Low educated can expect to live the fewest years in good health between ages 50 and 69 for the SAH indicator, followed by the OECD indicator, the OECD indicator without hearing and seeing and the ADL indicator. High educated can expect to live longer in good health for all indicators than low educated. Low educated men show a noticeable increase in years in good health only for the OECD indicator. For the other indicators the years in good health appear virtually constant. For high educated men, there is a noticeable increase over time for the years in good health for the OECD indicator. For the other indicators, the years in good health remain virtually constant.

High educated women also live more years in good health than low educated women for all indicators. Low educated women show a noticeable decrease in years in good health for the SAH indicator, the OECD indicator without hearing and seeing and for the ADL indicator. Low educated women are the only group with no increase in years in good health for the OECD indicator. High educated women experience a slight increase in years in good health for the SAH, OECD, OECD without hearing and seeing and the ADL indicator between 2006 and 2030.

Figure 2 and Additional file 1: Appendix 6 also show the partial life expectancy for ages 50–69. Life expectancy between age 50 and 69 is lower among the low educated as compared to the high educated and increases slightly in all groups, except for low educated women.

### Surplus and deficit of years in good health relative to the pension age

**Fig. 3:**
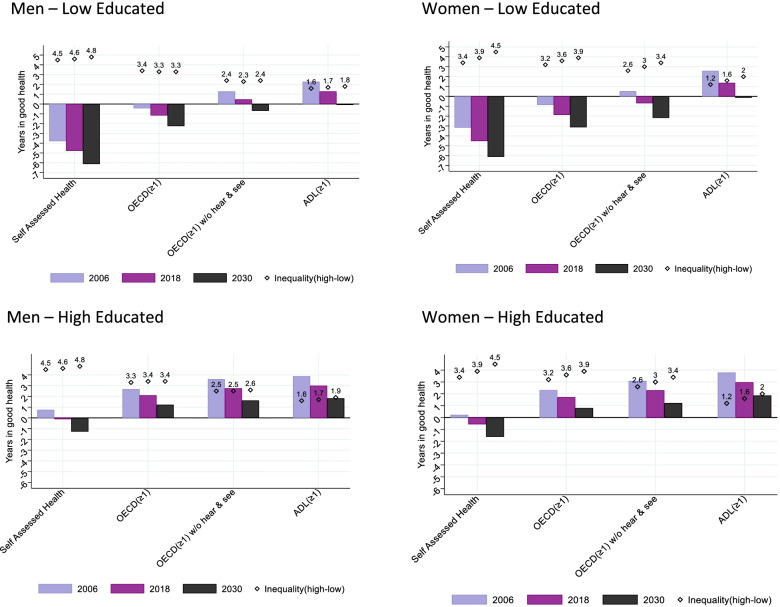
`Deficit’ and `Surplus’ of years in good health relative to the pension age for different health indicators for individuals between 50 and 69 by year, gender, education and related educational inequalities

Figure 3 shows the difference between the years in good health for each health indicator and the pension age for years 2006, 2018 and 2030, expressed as ‘deficit’ and ‘surplus’, by gender and education (for tables see Additional file 1: Appendix 7). It also shows the related absolute educational inequalities (low-high) in `deficit’ or `surplus’. Low educated men on average do not expect to reach the pension age in good health for any of the four indicators. For the SAH indicator, the period of good health is expected to end 6 years before retiring in 2030. This is 2 years for the OECD indicator, and 1 or less for the other indicators. For high educated men, the only indicator for which the period in good health is expected to end before the pension age in 2030 is SAH, with a deficit of around 1.2 years. For the other health indicators, high educated men are expected to have years left in good health at the pension age in 2030. The pattern is similar for women (See Additional file 1: Appendix 8 for medium educated).

There is no indication for a reduction in the gap between the low and high educated in the deficit/surplus for any of the indicators. For men, inequalities for the SAH indicator tend to increase slightly from 4.6 to 4.8 years between 2018 and 2030 and from 1.6 to 1.9 years for the ADL indicator. For the other indicators the inequalities are virtually constant. For women, the gap in the deficit/surplus between low and high educated was 3.9 years in 2018 and 4.5 years in 2030 for the SAH indicator. For the other indicators the increases were smaller (0.4 years).

Both for men and women, trends of poor health affected the increase in gap for deficit/surplus most (See Additional file 1: Appendix 9).

## Discussion

We find that for both genders, low educated not only have higher prevalence of poor health for each of the four health indicators than high educated, but also that over time the prevalences are increasing or flat at best for the low educated, while they are decreasing or flat for the high educated. The only exception is the OECD indicator, that appears to be decreasing over time for all education levels, except for low educated women. For low educated men, these prevalence trends, combined with the mortality trends, translate into increasing years in good health between ages 50 to 69 only for the OECD indicator, and constant years for the other indicators between 2006 and 2030. High educated men experience increasing years in good health only for OECD indicator and constant levels for the rest. Low educated women experience decreasing numbers of years in good health for three of the indicators, excluding the OECD indicator that is constant over time. High educated women experience a slightly increasing number of years in good health for the four indicators between 2006 and 2030. The changes over time were most unfavorable for low educated women.

Incorporating the increases in the statutory pension age over the 3 years in the analyses shows that low educated men and women are expected to have a `deficit’ of years in good health prior to the pension age for all four indicators by 2030, though with the ADL indicator being close to zero. The high educated, with the same increase in pension age, are expected to keep a surplus of years in good health after the pension age for most indicators, except for a small `deficit’ for SAH. Our results suggest a widening in the inequalities between high and low educated in the deficit/surplus for women for all indicators, and a slight widening for men but only for the SAH and ADL indicator.

### Prior research

To our knowledge there are no prior studies on deficit/surplus relative to the increasing pension age by education. The study of Majer et al. [4] examined socioeconomic inequalities in health expectancies between age 50 and 65 years in 10 Western-European countries for the period 1995–2001, but this was before the implementation of the policy change to increase the pension age. The study of Fontijn et al. [25] focused on the impact of the increasing pension age, however, it does not include different socioeconomic groups and does not provide insight in the size of the gap between the end of the healthy life and the revised pension age [23].

Several studies showed that increasing the statutory pension age increases the labour participation of older persons and the realised pension age [39], also in the Netherlands [40, 41]. There is less literature about differences between socioeconomic groups. In the United States, it was found that lower educated men delayed pensioning in response to an annual increase in pension in the period 2000–2006, but higher educated men and lower and higher educated women did not delay it [42]. In contrast, in the Netherlands between 2013 and 2018, the increase in realized pension age was larger for the low educated than for the high educated [43], but among low educated also the percentage spent with unemployment or disability benefits was a higher [44].

Increasing the pension age may also affect health. Prior studies provided conflicting evidence, with some studies finding improvements in health, and other not [45–48]. Two studies found increasing health inequalities between socio-economic groups [45, 46]. Our study does not take into account a possible causal effect of delaying the pension age on health.

### Interpretation

The analyses of `surplus’ and `deficit’ of years in good health relative to the pension age present an overview of the net effect of three parts. First, the prevalence of ill health (both levels and trends) determines the number of years expected to live in good health. Second, mortality impacts the number of years in good health. Third, the statutory pension age impacts the years in good health beyond (surplus) or below (deficit) this age. Educational differences and changes over time in the first two parts and uniform changes in the last part determine educational differences in surplus and deficits, and changes over time. In particular for women, changes in all three parts contribute to the increase in deficit of the low educated and increasing gaps as compared to high educated peers. The life expectancy between age 50 and 69 is expected to increase for high educated women but not for low educated women. This leads to around 20–25% of the increase in the surplus/deficit gap being due to these trends in mortality, and the rest due to trends in ill-health, since the pension age impacts both groups similarly. For men, the increase in life expectancy is similar for both low and high educated, and the slight increase in this gap is due to trends of poor health.

Several of the health indicators have been shown to increase the risk of premature labor market exit. Poor SAH has been found to impact early work exit in the Netherlands [11] and in several countries in Europe [7–12] and the United States [49]. Functional limitations (cutting toenails, dressing/undressing, walking steps, sitting down/getting up, use public transport) have been shown to have an impact on leaving work early due to disability pension in the Netherlands, and more so for the low and intermediate educated than for the high educated [7]. Evidence is mixed which indicator is most strongly associated with work. A recent study based on 11 European countries (including the Netherlands) indicates that poor SAH was more strongly associated with early exit from work due to disability benefits than other indicators such as chronic diseases, mobility limitations, and IADL-disability [9]. However, evidence from Spain indicates that disability measured with the Global Activity Limitation Indicator (GALI) reflected work activity better than SAH [50]. For this reason we presented several measures. It would have been desirable to have additionally included the GALI indicator, but it was only introduced recently in the survey and the question changed twice.

Several of the health indicators used in our study have been shown to increase the risk of labor market exit. Our findings of inequalities in the `surplus’ or `deficit’ of years in good health relative to the increasing pension age may therefore point at unequal chances to work until the pension age. The deficit of years in good health, however, should not be interpreted as years that an individual is unable to work, but as years when persons are at increased risk to leave employment because of health reasons. The strength of the association between employment exit and poor health is the product of complex interactions of individual-level factors (health status being the most important) [12], meso-level factors (e.g., workplace) and macro-level factors (e.g., social security arrangements, measures to keep persons at work). According to recent evidence, the working life expectancy of years 58-year old persons with disability was 1.5 years as compared to 5.5 years for all 58-year old persons in the Netherlands [51].

### Strengths and limitations

Strengths of this study include the use of a large number of cross-sectional waves of the Dutch Health Interview survey, spanning for a period of 29 years, and including four heath indicators exploring different aspects of health.

Some limitations of the study relate to our estimation of years in good health and resulting deficits and surpluses relative to the pension age. We obtained years in good health between age 50 and 69 based on the period Sullivan method. Our data did not allow us to use a cohort perspective. The period life expectancy in good health underestimates life expectancy in good health of cohorts in the case of decreasing mortality and/or decreasing prevalence of poor health over time. However, in our study which included a limited age and time range, differences are expected to be small. The Sullivan method, when using a period perspective, involves the stationary assumptions [52]. Simulation studies, however, have shown that these assumptions have minor influence on the results, unless large changes have occurred in mortality and/or disability in the study period [53–55]. Majer et al. [5] used a multistate life table approach to project health expectancy by education. However this study estimated transition probabilities between the health states and from each health state to death from different sources, which involved making additional assumptions.

For the calculation of the deficit, we assumed that years of good health occur before years in poor health. We focus on averages and ignored that at the individual level, individuals can cycle in and out of poor health and that at the group level some persons stay the entire time span in good health, while others are the entire timespan in poor health. Also we included the entire age group 65–69 in the calculation of years in good health, because we had data in 5-year age groups. This may have resulted in an underestimation of the deficit of years in good health.

In addition, some limitations relate to health indicators. Considering that trends of poor health account for most of the increasing trend in inequalities in the deficit or surplus of years in good health, our results are driven heavily by the estimated prevalence trends in the logistic models based on the health interview survey. The health indicators are self-reported and thus subject to heterogeneity in tendency to report health problems [56]. We expect that heterogeneity in reporting is less likely to affect trends. A more important uncertainty is that trends in poor health differ between surveys [57]. The health indicators in our study are based on response rates ranging between 60 and 65% [26, 27]. The method of collection of the survey data changed, from paper questionnaire by mail prior to 1990, Computer Assisted Personal Interviewing (CAPI) between 1990 and 2009, and a mixed-mode design from 2010 onwards.

Finally, socioeconomic position is a multi-faceted phenomenon that cannot be captured by education, as done in our study, nor by either occupation or income alone [58]. Taking into account the intersectionality was not possible with the available data but could provide additional insight in variations in the unequal consequences of increasing the pension age.

The findings of this study regarding the quantification of inequalities in deficit and surpluses may not be generalizable to other European countries considering that these outcomes are determined by the levels and trends of mortality and disability by education and age, and the pension age at the different time points, which all vary between countries.

## Conclusion and implications

Socio-economic inequalities in levels and trends of mortality and particularly in the prevalence of ill-health, combined with the increasing pension age impact the low educated more adversely than the high educated. If current trends continue, and pension age rises as planned, low educated individuals (particularly women) will experience more years of poor health prior to the pension age, and these inequalities in the `deficit’ or `surplus’ tend to increase over time.

From a policy perspective, in theory there are several paths that could help mitigate the asymmetric impact of an overall change in the statutory pension age on the different groups. An objective of policy could be to eliminate the educational inequalities or even more radically, to eliminate the health inequalities between educational groups. Less radically is targeting measures to prevent work-related disability (e.g. avoiding high physical demand), and measures to enable persons better to continue working with disability (e.g., allowing to work less hours and allow more flexibility in organizing the working day). Differentiation of the pension age by socio-economic position, or reducing the financial consequences of leaving paid work because of health reasons, are the most readily available policies that can reduce the asymmetric impact of increasing the pension age.

## Supplementary Information

**Additional file 1: Appendix Table 1**. Summary of construction of the education variable using the Dutch Health Interview survey (1989–2018). **Appendix Table 2**. Sample size for individuals aged 50–69 from the Dutch Health Survey (1989–2018), by gender and year. **Appendix 3**. Extrapolation of mortality rates for age groups 50–54, 55–59, 60–64, 65–69 by gender and education. **Appendix 4**. Test for non-linearity and inclusion of three way-interaction term. **Appendix Table 5**. Age standardized prevalence and predicted prevalence of several health indicators, by gender, education level and year. **Appendix Table 6.** Years in good health for several health indicators for individuals aged 50–69, by gender, education and year. **Appendix Table 7**. Surplus or deficit of years in good health relative to the statutory retirement age in the Netherlands and educational inequalities, by gender, education and year. **Appendix 8**. Partial Life expectancy, healthy life years and `deficit’ or `surplus’ for the medium educated between ages 50–69 by gender. **Appendix Table 9**. Robustness - `Deficit’ and `Surplus’ of years in good health relative to the retirement age for different health indicators for individuals between 50 and 69 by year, gender, education and related educational inequalities – Alternative scenarios.

## Data Availability

The data that support the findings of this study are available from the Dutch Statistical Agency (Centraal Bureau voor de Statistiek - CBS) but restrictions apply to the availability of these data, which were used under license for the current study, and so are not publicly available. Data are however available from the authors upon reasonable request and with permission of CBS. The Dutch health interview survey were obtained from: Centraal Bureau voor de Statistiek *Permanent Onderzoek van de Leefsituatie - POLS Gezondheid 1981–2009*. DANS. 10.17026/dans-zrm-7r4z Centraal Bureau voor de Statistiek (2010): *Gezondheidsenquête 2010, 2011*. DANS. 10.17026/dans-z93-mj8s Centraal Bureau voor de Statistiek (CBS) (2014): *Gezondheidsenquête 2012*. DANS. 10.17026/dans-zcc-5stc Centraal Bureau voor de Statistiek (CBS) (2014): *Gezondheidsenquête 2013*. DANS. 10.17026/dans-zdk-dwmn Centraal Bureau voor de Statistiek (CBS) (2014): *Gezondheidsenquête 2014*. DANS. 10.17026/dans-xcm-u69z Centraal Bureau voor de Statistiek (CBS) (2016): *Gezondheidsenquête 2015 – GECON 2015*. DANS. 10.17026/dans-xwr-m26w Centraal Bureau voor de Statistiek (CBS) (2016): *Gezondheidsenquête 2016 - GECON 2016*. DANS. 10.17026/dans-xxa-e3m7 Centraal Bureau voor de Statistiek (CBS) (2017): *Gezondheidsenquête 2017 - GECON 2017*. DANS. 10.17026/dans-xxd-j335 Centraal Bureau voor de Statistiek (CBS) (2018): *Gezondheidsenquête 2018 - GECON 2018*. DANS. 10.17026/dans-z5s-b7ve
