# Supplementary material for: Projecting years in good health between age 50–69 by education in the Netherlands until 2030 using several health indicators - an application in the context of a changing pension age
Source: BMC Public Health. 2022 Apr 29;22:859. doi: 10.1186/s12889-022-13223-8 (PMC9055744; doi:10.1186/s12889-022-13223-8)
Supplement: Supplementary file 1 — Additional file 1: Appendix Table 1. Summary of construction of the education variable using the Dutch Health Interview survey (1989–2018). Appendix Table 2. Sample size for individuals aged 50–69 from the Dutch Health Survey (1989–2018), by gender and year. Appendix 3. Extrapolation of mortality rates for age groups 50–54, 55–59, 60–64, 65–69 by gender and education. Appendix 4. Test for non-linearity and inclusion of three way-interaction term. Appendix Table 5. Age standardized prevalence and predicted prevalence of several health indicators, by gender, education level and year. Appendix Table 6. Years in good health for several health indicators for individuals aged 50–69, by gender, education and year. Appendix Table 7. Surplus or deficit of years in good health relative to the statutory retirement age in the Netherlands and educational inequalities, by gender, education and year. Appendix 8. Partial Life expectancy, healthy life years and `deficit’ or `surplus’ for the medium educated between ages 50–69 by gender. Appendix Table 9. Robustness - `Deficit’ and `Surplus’ of years in good health relative to the retirement age for different health indicators for individuals between 50 and 69 by year, gender, education and related educational inequalities – Alternative scenarios. [file 12889_2022_13223_MOESM1_ESM.docx]

**Electronic Supplementary Material**

**Appendix Table 1. Summary of construction of the education variable using the Dutch Health Interview survey (1989-2018)**

| Period | Variable name | Education level | Level |  |
| --- | --- | --- | --- | --- |
| 1990-1996 | opleid | Low | Lager onderwijs | |
|  |  |  | LAVO-MAVO-LBO | |
|  |  |  |  | |
|  |  | Medium | HAVO-MBO | |
|  |  |  |  |  |
|  |  | High | HBO kandidaats | |
|  |  |  | Universiteit | |
|  |  |  |  | |
| 1997-2008 | vltoplop | Low | Lager onderwijs | |
|  |  |  | LBO |  |
|  |  |  | MAVO, VWO-3 | |
|  |  |  |  | |
|  |  | Medium | HAVO, VWO, MBO | |
|  |  |  |  |  |
|  |  | High | HBO, universiteit | |
|  |  |  |  | |
| 2009-2018 | oplniv5 | Low | Basisonderwijs | |
|  |  |  | VMBO, MBO 1, AVO, ONDERBOUW | |
|  |  |  |  |  |
|  |  | Medium | HAVO, VWO, MBO 2-4, MBO | |
|  |  |  |  |  |
|  |  | High | HBO, WO bachelor | |
|  |  |  | WO master, doctor | |

**Appendix Table 2. Sample size for individuals aged 50-69 from the Dutch Health Survey (1989-2018), by gender and year**

| \| **Year** \| **Men** \| **Women** \| **Total** \| \| --- \| --- \| --- \| --- \| \| 1989 \| 779 \| 855 \| 1634 \| \| 1990 \| 728 \| 764 \| 1492 \| \| 1991 \| 674 \| 756 \| 1430 \| \| 1992 \| 821 \| 883 \| 1704 \| \| 1993 \| 806 \| 879 \| 1685 \| \| 1994 \| 798 \| 872 \| 1670 \| \| 1995 \| 898 \| 966 \| 1864 \| \| 1996 \| 886 \| 924 \| 1810 \| \| 1997 \| 1064 \| 1025 \| 2089 \| \| 1998 \| 906 \| 927 \| 1833 \| \| 1999 \| 1014 \| 1050 \| 2064 \| \| 2000 \| 1046 \| 1046 \| 2092 \| \| 2001 \| 1022 \| 967 \| 1989 \| \| 2002 \| 1046 \| 1064 \| 2110 \| \| 2003 \| 1114 \| 1121 \| 2235 \| \| 2004 \| 1251 \| 1234 \| 2485 \| \| 2005 \| 1225 \| 1211 \| 2436 \| \| 2006 \| 1175 \| 1155 \| 2330 \| \| 2007 \| 1014 \| 1050 \| 2064 \| \| 2008 \| 1141 \| 1162 \| 2303 \| \| 2009 \| 686 \| 700 \| 1386 \| \| 2010 \| 2046 \| 2040 \| 4086 \| \| 2011 \| 1827 \| 1956 \| 3783 \| \| 2012 \| 1973 \| 1952 \| 3925 \| \| 2013 \| 1885 \| 1993 \| 3878 \| \| 2014 \| 1268 \| 1358 \| 2626 \| \| 2015 \| 1314 \| 1303 \| 2617 \| \| 2016 \| 1275 \| 1275 \| 2550 \| \| 2017 \| 1347 \| 1382 \| 2729 \| \| 2018 \| 1531 \| 1449 \| 2980 \| |  |  |
| --- | --- | --- | --- | --- | --- | --- | --- | --- | --- | --- | --- | --- | --- | --- | --- | --- | --- | --- | --- | --- | --- | --- | --- | --- | --- | --- | --- | --- | --- | --- | --- | --- | --- | --- | --- | --- | --- | --- | --- | --- | --- | --- | --- | --- | --- | --- | --- | --- | --- | --- | --- | --- | --- | --- | --- | --- | --- | --- | --- | --- | --- | --- | --- | --- | --- | --- | --- | --- | --- | --- | --- | --- | --- | --- | --- | --- | --- | --- | --- | --- | --- | --- | --- | --- | --- | --- | --- | --- | --- | --- | --- | --- | --- | --- | --- | --- | --- | --- | --- | --- | --- | --- | --- | --- | --- | --- | --- | --- | --- | --- | --- | --- | --- | --- | --- | --- | --- | --- | --- | --- | --- | --- | --- | --- | --- | --- |

**Appendix 3- Extrapolation of mortality rates for age groups 50-54, 55-59, 60-64, 65-69 by gender and education**

**Approach:**

This appendix gives a summary of the projection approach we used to forecast mortality rates by age, gender and education. Detailed information on the estimation and extrapolation approach can be found in [[1](#_ENREF_1)].

To model Dutch education-specific mortality, we use a three-layer Li and Lee model. The upper layer, labelled as ‘HMD’ models a common trend of all educational groups and countries. The second layer, labelled as ‘INT EDU’, models the deviation of education-specific mortality of selected European countries from the common trend. The third layer, the NL EDU layer, models the deviation of Dutch education-specific mortality from international education-specific mortality of the selected countries. Hence, Dutch education-specific mortality is the sum of the three layers.

Each of the three layers is modeled using the Lee and Carter approach [[1](#_ENREF_1)]. The Lee-Carter model summarizes mortality by age and period for a single population as a function of an age-effect, a time trend, and age-specific sensitivities to the time trend. [[2](#_ENREF_2)]. Similar to recent national projections in the Netherlands [[3](#_ENREF_3), [4](#_ENREF_4)], we use the Lee and Li approach [[5](#_ENREF_5)] multi-population extension to include mortality data from neighboring countries to increase the robustness of the projections. Whereas the original Li and Lee approach had two layers, our model has three layers to include also different educational groups. The three layer setup enables us to include extra information on national and education-specific mortality rates from five countries to derive a more stable and robust long-term trend for the Netherlands.

We estimated the three-layer Li and Lee model separately for men and women for the age range 35-80 years. In this publication we only use ages between 50 and 70 years.

**Data:**

The upper layer, ‘HMD’ uses national mortality data by 5 year age (35-39, 40-44, …., 80-84) and gender from the Human Mortality Database [[6](#_ENREF_6)] for the years 1970-2018.

The second layer ‘INT EDU’ uses an aggregate of mortality data by 5-year age, gender and education for the five North-Western European countries (Belgium, Switzerland, Denmark, Norway and Finland) for the 5-year periods between 1990-2014. The mortality data by education derived from a longitudinal mortality follow-up by Erasmus MC, see table below, used in prior publications [[7](#_ENREF_7), [8](#_ENREF_8)]. The five countries were selected because these countries provided data based on individual mortality follow up, had sufficiently long time series, provided data related to national populations and included three similar educational groups (low, mid and high educated). We excluded countries meeting these criteria with large educational inequalities (in Eastern and Central Europe) and small educational inequalities (in Southern Europe).

**Table A3 European mortality data by gender, age, education**

| **Country** | **Type** | **Years** | **Census date** | **Length of follow-up (yr)** | **Geographic coverage** |
| --- | --- | --- | --- | --- | --- |
| Belgium | Longitudinal | March 1 1990 – Dec 31, 1997 | 1.3.1997 | 6 Y, 10 M | National |
|  |  | Oct, 1 2001 – Dec 31, 2011 | 1.19.2001 | 10, 3 M | National |
| Denmark | Longitudinal | Jan 1, 1995- Dec 31 1999 | 1995 | 5 | National |
|  |  | Jan 1, 2000- Dec 31 2004 | 2000 | 5 | National |
|  |  | Jan 1, 2005- Dec 31 2009 | 2005 | 5 | National |
|  |  | Jan 1 2010- 3Dec 31 2014 | 2010 | 5 | National |
|  |  | March 2011-December 2013 | March 2011 | 2 Y, 10 M | National |
| Finland | Longitudinal | Dec 31, 1990 – Dec 31, 2000 | 31.12.1990 | 10 | National |
|  |  | Dec 31, 2000 – Dec 31, 2010 | 31.12.2000 | 10 | National |
|  |  | Dec 31, 2010 –.Dec 31, 2014 | 31.12.2010 | 4 | National |
| Norway | Longitudinal | Nov, 1990 – Dec, 2001 | Nov, 1990 | 11 | National |
|  |  | Nov, 2001 – Dec, 2009 | Nov, 2001 | 8 | National |
|  |  | Jan 2000 – Dec 2008 | 1.2001 | 9 | National |
| Switzerland | Longitudinal | Dec 4, 1990 – Dec 5, 2000 | 4.12.1990 | 10 | National |
|  |  | Dec 31, 2000 – Dec 31, 2010 | 5.12.2000 | 10 | National |
|  |  | Dec 31 2010 – Dec 31, 2014 | 31.12.2010 | 4 | National |

The third layer, NL-EDU’ uses yearly mortality rates for the Netherlands, by education, 5- year age group, gender and single calendar year for the period 2006-2018 derived within the secure environment of Statistical Netherlands. This is based on an individual linkage of educational register, mortality register and population register.

**Table B3 Summary of total number of deaths and person years in the mortality data**

| **Country** | **Education** | **Deaths** | **Person Years** |
| --- | --- | --- | --- |
| Belgium | Low | 1165334 | 51038290 |
| Belgium | Mid | 177225 | 21504660 |
| Belgium | High | 118836 | 18624384 |
| Denmark | Low | 393680 | 20345764 |
| Denmark | Mid | 228399 | 25201252 |
| Denmark | High | 85028 | 15028869 |
| Finland | Low | 1587379 | 62060261 |
| Finland | Mid | 255404 | 30618591 |
| Finland | High | 168860 | 24107525 |
| Netherlands | Low | 115021 | 14912715 |
| Netherlands | Mid | 72196 | 21782186 |
| Netherlands | High | 37231 | 20698799 |
| Norway | Low | 992196 | 29689344 |
| Norway | Mid | 477520 | 25580187 |
| Norway | High | 96907 | 8631005 |
| Switzerland | Low | 587125 | 20831871 |
| Switzerland | Mid | 478580 | 41367716 |
| Switzerland | High | 119882 | 14022826 |

**Pre-processing steps**

To accommodate differences in length of the historical time series and level of granulation of the period data between the different countries in layer 2, we selected common mid-points: 1993, 1998, 2003, 2008 and 2013 to minimize changes in the composition of the group of countries over time. In cases where a midpoint did not match exactly with the common midpoint, we shifted the midpoint to the common midpoint (e.g. for Belgium the calculated mid-point was 1994 and this was shifted to 1993). The shifts were maximum 1 year.

**Estimation method Li & Lee model:**

We used the modified estimation method of Liu et al [9] to estimate the parameters of the three layers in the Li and Lee model. To avoid strong assumptions, we did not assume common trends for all educational groups, nor convergence to a common trend. The time-series processes for the time trends in the three layers are selected based on visual inspection of aggregate (over age groups) observed mortality rates over time. For HMD, a random walk with drift (RWD) was selected because the data shows a clear and stable declining trend over time. For the deviation between international education-specific mortality and HMD mortality (layer 2), we selected a RWD process for the low and high educated, and an AR1 (autoregressive model of order 1) process for the mid educated an (ARIMA 0,1,0) here: AR1). The reason is that the data suggests divergence from the common trend for the low and high educated (hence, an RWD), but not for the mid educated. The time series for the time trend in the third layer (NL EDU) is modeled as a constant plus random noise (an ARIMA (0,0,0) with constant) because the data suggests that the difference between Dutch education-specific mortality and international education-specific mortality fluctuates around a constant.

To cover the same period and level of granulation as in the third Dutch layer, we used interpolation/extrapolation in the first two layers. In layer 1, the estimated parameters of the time series process in layer one were used to extrapolate HMD mortality rates to the years 2017 and 2018. In layer 2, the estimated parameters of the time series process were used to interpolate mortality rates to single years between the common midpoints for the period 1993 till 2013, and to extrapolate to single years for the period 2014 till 2018.

**Table C3. Mortality rates by age, gender and education**

| **Men** |  | Year | | |
| --- | --- | --- | --- | --- |
| Age | Education | 2006 | 2018 | 2030 |
| 50-54 | Low | 0.005454 | 0.004611 | 0.004282 |
| 55-59 | Low | 0.008624 | 0.007687 | 0.007191 |
| 60-64 | Low | 0.014118 | 0.011810 | 0.010609 |
| 65-69 | Low | 0.022092 | 0.017964 | 0.015496 |
| 50-54 | Med | 0.003927 | 0.003015 | 0.002297 |
| 55-59 | Med | 0.006428 | 0.004799 | 0.003812 |
| 60-64 | Med | 0.011143 | 0.008311 | 0.006212 |
| 65-69 | Med | 0.017995 | 0.013153 | 0.009869 |
| 50-54 | High | 0.002385 | 0.001960 | 0.001523 |
| 55-59 | High | 0.004140 | 0.003519 | 0.002943 |
| 60-64 | High | 0.007080 | 0.005461 | 0.004434 |
| 65-69 | High | 0.012585 | 0.009527 | 0.007443 |
| **Women** |  |  |  |  |
| 50-54 | Low | 0.003624 | 0.003879 | 0.004111 |
| 55-59 | Low | 0.005278 | 0.005725 | 0.006126 |
| 60-64 | Low | 0.007778 | 0.007906 | 0.007972 |
| 65-69 | Low | 0.011515 | 0.010799 | 0.010165 |
| 50-54 | Med | 0.002695 | 0.002319 | 0.001961 |
| 55-59 | Med | 0.003914 | 0.003386 | 0.002874 |
| 60-64 | Med | 0.006123 | 0.005351 | 0.004409 |
| 65-69 | Med | 0.009017 | 0.007757 | 0.006067 |
| 50-54 | High | 0.001870 | 0.001555 | 0.001341 |
| 55-59 | High | 0.003070 | 0.002615 | 0.002300 |
| 60-64 | High | 0.004540 | 0.003839 | 0.003280 |
| 65-69 | High | 0.007556 | 0.006307 | 0.005125 |

1. Lee, R.D. and L.R. Carter, *Modelling and Forecasting U.S. Mortality.* Journal of the American Statistical Association, 1992. **87**: p. 659-671.

2. Janssen, F., *Advances in mortality forecasting: introduction.* Genus, 2018. **74**(1): p. 21.

3. Stoeldraijer, L., et al., *Impact of different mortality forecasting methods and explicit assumptions on projected future life expectancy: The case of the Netherlands.* Demographic Research, 2013. **29**: p. 323-354.

4. Li, N. and R. Lee, *Coherent mortality forecasts for a group of populations: An extension of the Lee-Carter method.* Demography, 2005. **42**(3): p. 575-594.

5. Tilburg en EMC, *The future of life expectancy by education in the Netherlands (of andere titel).* submitted, 2021.

6. University of California, B.U., and Max Planck Institute for Demographic Research (Germany). . *Human Mortality Database* 2020 [cited 2020; Available from: [www.mortality.org](file:///\\storage.erasmusmc.nl\v\vcl13\MAGE\DATA\UserData\611645\AaWilma-arch\Projecten\Netspar\Projections%20by%20education\www.mortality.org).

7. Mackenbach, J.P., et al., *Progress against inequalities in mortality: register-based study of 15 European countries between 1990 and 2015.* Eur J Epidemiol, 2019.

8. Mackenbach, J.P., et al., *Trends in health inequalities in 27 European countries.* Proc Natl Acad Sci U S A, 2018. **115**(25): p. 6440-6445.

9. Liu, Q., C. Ling, and L. Peng, *Statistical Inference for Lee-Carter Mortality Model and Corresponding Forecasts.* North American Actuarial Journal, 2019. **23**(3): p. 335-363.

**Appendix 4.– Test for non-linearity and inclusion of three way-interaction term**

**Appendix Table 4.a Likelihood ratio tests and Akaike Information criterium (AIC) comparison between the main specification and alternative specification (cubic splines model) for different health indicators for individuals between 50-69 by gender**

Lowest AIC in gray

| **Men** |  |  |  |  |
| --- | --- | --- | --- | --- |
|  | **SAH** | **OECD** | **OECD w/o hear and see** | **ADL** |
| **Model** | AIC | AIC | AIC | AIC |
| Main model | 40120 | 22849 | 14424 | 9548 |
| Cubic spline model | 40117 | 22849 | 14402 | 9553 |
| *LR test (p-value)** | *0.05* | *0.11* | *0.00* | *0.32* |
|  |  |  |  |  |
| **Women** |  |  |  |  |
|  | **SAH** | **OECD** | **OECD w/o hear and see** | **ADL** |
| **Model** | AIC | AIC | AIC | AIC |
| Main model | 43268 | 27774 | 22761 | 13087 |
| Cubic Splines model | 43272 | 27758 | 22734 | 13090 |
| *LR test (p-value)** | *0.59* | *0.00* | *0.00* | *0.30* |

The logistic models included age category (50-54;..;65-69), education level (low, medium, high), year of the survey, and interaction term between education and year. Comparison includes age category, education level, cubic splines for year, interaction of splines with education. Health indicators included are: less than good SAH(SAH), Organization for Economic Cooperation and Development (OECD) functional limitation indicator, OECD indicator without hearing and seeing items, and Activities of Daily living (ADLs).

We estimated models using cubic splines for year and used a likelihood ratio test (LR test) between this model and our main specification to test for non-linearities.* P-value below 0.05 means spline model is better fit.

| **Men** |  |  |  |  |
| --- | --- | --- | --- | --- |
|  | **SAH** | **OECD** | **OECD w/o hear and see** | **ADL** |
| **Model** | AIC | AIC | AIC | AIC |
| Main model | 40120 | 22849 | 14424 | 9548 |
| Interaction (age*educ*year) | 40118 | 22854 | 14428 | 9553 |
| LR test (p-val) | 0.01 | 0.06 | 0.06 | 0.14 |
|  |  |  |  |  |
| **Women** |  |  |  |  |
|  | **SAH** | **OECD** | **OECD w/o hear and see** | **ADL** |
| **Model** | AIC | AIC | AIC | AIC |
| Main model | 43268 | 27774 | 22761 | 13087 |
| Interaction (age*educ*year) | 43257 | 27758 | 22738 | 13087 |
| LR Test (p-val) | 0.00 | 0.00 | 0.00 | 0.05 |

**Appendix Table 4.b Robustness – Akaike Information criterium (AIC) comparison between the main specification and alternative specification (three-way interaction age category* education*year) for different health indicators for individuals between 50-69 by gender**

Lowest AIC in gray

The logistic models included age category (50-54;..;65-69), education level (low, medium, high), year of the survey, and interaction term between education and year. Comparison includes three way interaction between age, education and year. Health indicators included are: less than good SAH(SAH), Organization for Economic Cooperation and Development (OECD) functional limitation indicator, OECD indicator without hearing and seeing items, and Activities of Daily living (ADLs)

We estimated models using three way interaction education*agecat year and used a likelihood ratio test (LR test) between this model and our main specification. P-value below 0.05 means 3 way interaction model is a better fit model is better fit.

**Appendix Table 4.c - Robustness - Years in good health relative to the retirement age for different health indicators for individuals between 50-69 by year, gender, education– Alternative specification (interaction age category* education*year)**

**Low Educated Men Low Educated Women**

Main Results

Main Results

Includes interaction term (agecat*educ*year)

Includes interaction term (agecat*educ*year)

**High Educated Men High Educated Women**

Includes interaction term (agecat*educ*year)

Main Results

Main Results

Includes interaction term (agecat*educ*year)

Years in good health obtained from combining the predicted prevalences by logistic models from the Dutch Health Interview Survey and projected mortality rates

The logistic models included age category (50-54;..;65-69), education level (low, medium, high), year of the survey, and interaction term between education and year. Comparison includes three way interaction between age, education and year. Health indicators included are: less than good SAH(SAH), Organization for Economic Cooperation and Development (OECD) functional limitation indicator, OECD indicator without hearing and seeing items, and Activities of Daily living (ADLs)

**Appendix Table 4.d - `Deficit’ and `Surplus’ of years in good health relative to the retirement age for different health indicators for individuals between 50-69 by year, gender, education and related educational inequalities - Alternative specification (age category* education*year)**

**Low Educated Men Low Educated Women**

Main Results

Main Results

Includes interaction term (agecat*educ*year)

Includes interaction term (agecat*educ*year)

**High Educated Men High Educated Women**

Main Results

Main Results

Includes interaction term (agecat*educ*year)

Includes interaction term (agecat*educ*year)

The difference between years in good health for each health indicator and the years remaining to the pension age from age 50 at these three points in time: (Years in good health between ages 50 and 70– (pension age-50)). In 2006 the pension age was 65 years, in 2018 it was 66 years and in 2030 it will be 67 years and 3 months. We present the `deficit’ of years in good health to reach the pension age in good health or `surplus’ of years in good health expected to live past the pension age. Health indicators included are: less than good SAH(SAH), Organization for Economic Cooperation and Development (OECD) functional limitation indicator, OECD indicator without hearing and seeing items, and Activities of Daily living (ADLs)

| **Appendix Table 5. Age standardized prevalence and predicted prevalence of several health indicators, by gender, education level and year** |
| --- |

|  |  | **Age Standardized Prevalence^a^** | | | **Model Predicted Prevalence^b^** | | |
| --- | --- | --- | --- | --- | --- | --- | --- |
| **Year** | **Health Indicator** | **L** | **M** | **H** | **L** | **M** | **H** |
| 1989 | SRH LG | 41.2 | 23.3 | 22.3 | 37.7 | 27.9 | 17.5 |
| 1990 | SRH LG | 44.3 | 32.2 | 14.1 | 37.7 | 27.8 | 17.5 |
| 1991 | SRH LG | 40.1 | 33.6 | 22.7 | 37.8 | 27.7 | 17.5 |
| 1992 | SRH LG | 46.3 | 24.9 | 14.0 | 37.8 | 27.6 | 17.5 |
| 1993 | SRH LG | 38.2 | 29.6 | 15.4 | 37.9 | 27.5 | 17.5 |
| 1994 | SRH LG | 38.4 | 28.9 | 19.2 | 37.9 | 27.4 | 17.5 |
| 1995 | SRH LG | 37.7 | 27.8 | 20.2 | 38.0 | 27.3 | 17.5 |
| 1996 | SRH LG | 36.7 | 24.0 | 17.6 | 38.0 | 27.2 | 17.5 |
| 1997 | SRH LG | 38.4 | 25.6 | 15.8 | 38.1 | 27.1 | 17.5 |
| 1998 | SRH LG | 34.8 | 26.8 | 13.9 | 38.2 | 27.0 | 17.5 |
| 1999 | SRH LG | 33.8 | 27.0 | 17.7 | 38.2 | 26.9 | 17.5 |
| 2000 | SRH LG | 35.4 | 28.2 | 17.3 | 38.3 | 26.8 | 17.5 |
| 2001 | SRH LG | 34.3 | 23.7 | 19.3 | 38.3 | 26.7 | 17.5 |
| 2002 | SRH LG | 38.5 | 30.8 | 22.4 | 38.4 | 26.6 | 17.5 |
| 2003 | SRH LG | 35.0 | 26.5 | 19.8 | 38.4 | 26.5 | 17.5 |
| 2004 | SRH LG | 44.1 | 24.6 | 18.3 | 38.5 | 26.4 | 17.5 |
| 2005 | SRH LG | 39.6 | 27.1 | 17.5 | 38.5 | 26.3 | 17.6 |
| 2006 | SRH LG | 36.9 | 24.1 | 14.9 | 38.6 | 26.2 | 17.6 |
| 2007 | SRH LG | 38.1 | 25.6 | 11.6 | 38.6 | 26.1 | 17.6 |
| 2008 | SRH LG | 36.7 | 24.5 | 17.6 | 38.7 | 26.0 | 17.6 |
| 2009 | SRH LG | 38.1 | 20.6 | 10.0 | 38.7 | 25.9 | 17.6 |
| 2010 | SRH LG | 36.7 | 28.3 | 16.6 | 38.8 | 25.8 | 17.6 |
| 2011 | SRH LG | 39.5 | 26.7 | 17.4 | 38.9 | 25.7 | 17.6 |
| 2012 | SRH LG | 36.4 | 26.5 | 19.2 | 38.9 | 25.7 | 17.6 |
| 2013 | SRH LG | 43.7 | 29.2 | 17.2 | 39.0 | 25.6 | 17.6 |
| 2014 | SRH LG | 35.5 | 25.3 | 19.0 | 39.0 | 25.5 | 17.6 |
| 2015 | SRH LG | 40.9 | 24.5 | 17.5 | 39.1 | 25.4 | 17.6 |
| 2016 | SRH LG | 39.2 | 21.4 | 17.6 | 39.1 | 25.3 | 17.6 |
| 2017 | SRH LG | 43.9 | 25.7 | 19.8 | 39.2 | 25.2 | 17.6 |
| 2018 | SRH LG | 45.3 | 27.4 | 17.7 | 39.2 | 25.1 | 17.6 |
| 2019 | SRH LG |  |  |  | 39.3 | 25.0 | 17.6 |
| 2020 | SRH LG |  |  |  | 39.3 | 24.9 | 17.6 |
| 2021 | SRH LG |  |  |  | 39.4 | 24.8 | 17.6 |
| 2022 | SRH LG |  |  |  | 39.5 | 24.7 | 17.6 |
| 2023 | SRH LG |  |  |  | 39.5 | 24.6 | 17.6 |
| 2024 | SRH LG |  |  |  | 39.6 | 24.5 | 17.6 |
| 2025 | SRH LG |  |  |  | 39.6 | 24.4 | 17.6 |
| 2026 | SRH LG |  |  |  | 39.7 | 24.3 | 17.6 |
| 2027 | SRH LG |  |  |  | 39.7 | 24.3 | 17.6 |
| 2028 | SRH LG |  |  |  | 39.8 | 24.2 | 17.7 |
| 2029 | SRH LG |  |  |  | 39.8 | 24.1 | 17.7 |
| 2030 | SRH LG |  |  |  | 39.9 | 24.0 | 17.7 |
| 1989 | OECD (≥1) | 23.2 | 11.9 | 11.4 | 23.6 | 15.9 | 10.5 |
| 1990 | OECD (≥1) | 29.6 | 18.1 | 8.9 | 23.5 | 15.7 | 10.3 |
| 1991 | OECD (≥1) | 25.0 | 14.6 | 10.8 | 23.4 | 15.5 | 10.1 |
| 1992 | OECD (≥1) | 23.9 | 14.9 | 10.2 | 23.2 | 15.3 | 9.9 |
| 1993 | OECD (≥1) | 28.0 | 14.3 | 9.8 | 23.1 | 15.1 | 9.8 |
| 1994 | OECD (≥1) | 28.2 | 20.1 | 9.8 | 23.0 | 14.9 | 9.6 |
| 1995 | OECD (≥1) | 26.0 | 21.1 | 7.5 | 22.9 | 14.8 | 9.4 |
| 1996 | OECD (≥1) | 22.8 | 11.9 | 12.4 | 22.8 | 14.6 | 9.3 |
| 1997 | OECD (≥1) | 19.0 | 12.1 | 13.1 | 22.7 | 14.4 | 9.1 |
| 1998 | OECD (≥1) | 18.7 | 12.0 | 6.5 | 22.6 | 14.3 | 8.9 |
| 1999 | OECD (≥1) | 19.5 | 12.8 | 6.2 | 22.5 | 14.1 | 8.8 |
| 2000 | OECD (≥1) | 18.5 | 14.1 | 10.6 | 22.4 | 13.9 | 8.6 |
| 2001 | OECD (≥1) | 18.9 | 9.6 | 6.9 | 22.3 | 13.7 | 8.5 |
| 2002 | OECD (≥1) | 20.3 | 16.2 | 7.3 | 22.2 | 13.6 | 8.3 |
| 2003 | OECD (≥1) | 25.0 | 11.1 | 9.1 | 22.1 | 13.4 | 8.2 |
| 2004 | OECD (≥1) | 23.5 | 12.3 | 10.6 | 22.0 | 13.3 | 8.0 |
| 2005 | OECD (≥1) | 19.5 | 16.0 | 10.1 | 21.9 | 13.1 | 7.9 |
| 2006 | OECD (≥1) | 23.6 | 12.1 | 6.9 | 21.8 | 12.9 | 7.7 |
| 2007 | OECD (≥1) | 22.9 | 17.9 | 3.3 | 21.7 | 12.8 | 7.6 |
| 2008 | OECD (≥1) | 17.7 | 11.4 | 7.3 | 21.5 | 12.6 | 7.5 |
| 2009 | OECD (≥1) | 24.3 | 12.0 | 7.8 | 21.4 | 12.5 | 7.3 |
| 2010 | OECD (≥1) | 26.2 | 14.1 | 11.7 | 21.3 | 12.3 | 7.2 |
| 2011 | OECD (≥1) | 22.6 | 15.9 | 10.8 | 21.2 | 12.2 | 7.1 |
| 2012 | OECD (≥1) | 20.5 | 14.6 | 8.7 | 21.1 | 12.0 | 6.9 |
| 2013 | OECD (≥1) | 21.6 | 17.5 | 8.6 | 21.0 | 11.9 | 6.8 |
| 2014 | OECD (≥1) | 19.9 | 11.2 | 5.8 | 20.9 | 11.7 | 6.7 |
| 2015 | OECD (≥1) | 23.6 | 12.3 | 4.6 | 20.8 | 11.6 | 6.6 |
| 2016 | OECD (≥1) | 19.4 | 10.3 | 7.0 | 20.7 | 11.4 | 6.5 |
| 2017 | OECD (≥1) | 20.9 | 9.5 | 4.3 | 20.6 | 11.3 | 6.3 |
| 2018 | OECD (≥1) | 23.4 | 10.2 | 6.8 | 20.5 | 11.2 | 6.2 |
| 2019 | OECD (≥1) |  |  |  | 20.4 | 11.0 | 6.1 |
| 2020 | OECD (≥1) |  |  |  | 20.3 | 10.9 | 6.0 |
| 2021 | OECD (≥1) |  |  |  | 20.2 | 10.8 | 5.9 |
| 2022 | OECD (≥1) |  |  |  | 20.1 | 10.6 | 5.8 |
| 2023 | OECD (≥1) |  |  |  | 20.0 | 10.5 | 5.7 |
| 2024 | OECD (≥1) |  |  |  | 19.9 | 10.4 | 5.6 |
| 2025 | OECD (≥1) |  |  |  | 19.8 | 10.2 | 5.5 |
| 2026 | OECD (≥1) |  |  |  | 19.7 | 10.1 | 5.4 |
| 2027 | OECD (≥1) |  |  |  | 19.7 | 10.0 | 5.3 |
| 2028 | OECD (≥1) |  |  |  | 19.6 | 9.9 | 5.2 |
| 2029 | OECD (≥1) |  |  |  | 19.5 | 9.7 | 5.1 |
| 2030 | OECD (≥1) |  |  |  | 19.4 | 9.6 | 5.0 |
| 1989 | OECD (v2) | 14.8 | 6.5 | 5.4 | 11.1 | 6.7 | 2.7 |
| 1990 | OECD (v2) | 17.7 | 13.4 | 2.5 | 11.2 | 6.7 | 2.7 |
| 1991 | OECD (v2) | 12.5 | 7.4 | 4.0 | 11.2 | 6.6 | 2.7 |
| 1992 | OECD (v2) | 12.1 | 6.6 | 1.9 | 11.2 | 6.6 | 2.7 |
| 1993 | OECD (v2) | 18.6 | 4.1 | 5.3 | 11.3 | 6.6 | 2.7 |
| 1994 | OECD (v2) | 10.9 | 8.6 | 3.3 | 11.3 | 6.6 | 2.7 |
| 1995 | OECD (v2) | 11.3 | 10.1 | 2.8 | 11.4 | 6.6 | 2.7 |
| 1996 | OECD (v2) | 11.7 | 5.6 | 5.8 | 11.4 | 6.5 | 2.7 |
| 1997 | OECD (v2) | 9.1 | 6.3 | 5.5 | 11.4 | 6.5 | 2.7 |
| 1998 | OECD (v2) | 8.0 | 4.6 | 2.4 | 11.5 | 6.5 | 2.7 |
| 1999 | OECD (v2) | 9.9 | 6.2 | 2.4 | 11.5 | 6.5 | 2.7 |
| 2000 | OECD (v2) | 9.3 | 6.1 | 3.1 | 11.6 | 6.4 | 2.7 |
| 2001 | OECD (v2) | 8.1 | 5.1 | 0.8 | 11.6 | 6.4 | 2.8 |
| 2002 | OECD (v2) | 9.9 | 7.6 | 0.9 | 11.6 | 6.4 | 2.8 |
| 2003 | OECD (v2) | 12.4 | 5.3 | 2.2 | 11.7 | 6.4 | 2.8 |
| 2004 | OECD (v2) | 12.2 | 5.2 | 2.8 | 11.7 | 6.4 | 2.8 |
| 2005 | OECD (v2) | 10.3 | 5.3 | 2.5 | 11.8 | 6.3 | 2.8 |
| 2006 | OECD (v2) | 13.3 | 3.5 | 1.6 | 11.8 | 6.3 | 2.8 |
| 2007 | OECD (v2) | 11.3 | 7.6 | 1.3 | 11.9 | 6.3 | 2.8 |
| 2008 | OECD (v2) | 7.6 | 4.8 | 1.4 | 11.9 | 6.3 | 2.8 |
| 2009 | OECD (v2) | 12.5 | 2.8 | 3.3 | 11.9 | 6.3 | 2.8 |
| 2010 | OECD (v2) | 12.4 | 6.1 | 3.7 | 12.0 | 6.2 | 2.8 |
| 2011 | OECD (v2) | 10.5 | 4.7 | 3.2 | 12.0 | 6.2 | 2.8 |
| 2012 | OECD (v2) | 8.0 | 5.7 | 2.0 | 12.1 | 6.2 | 2.8 |
| 2013 | OECD (v2) | 7.9 | 8.3 | 2.9 | 12.1 | 6.2 | 2.8 |
| 2014 | OECD (v2) | 15.1 | 8.4 | 3.0 | 12.1 | 6.2 | 2.8 |
| 2015 | OECD (v2) | 14.8 | 7.0 | 2.3 | 12.2 | 6.1 | 2.8 |
| 2016 | OECD (v2) | 14.8 | 6.3 | 3.6 | 12.2 | 6.1 | 2.8 |
| 2017 | OECD (v2) | 12.8 | 5.5 | 2.8 | 12.3 | 6.1 | 2.8 |
| 2018 | OECD (v2) | 17.0 | 6.6 | 4.1 | 12.3 | 6.1 | 2.8 |
| 2019 | OECD (v2) | |  |  | 12.4 | 6.1 | 2.9 |
| 2020 | OECD (v2) | |  |  | 12.4 | 6.0 | 2.9 |
| 2021 | OECD (v2) | |  |  | 12.4 | 6.0 | 2.9 |
| 2022 | OECD (v2) | |  |  | 12.5 | 6.0 | 2.9 |
| 2023 | OECD (v2) | |  |  | 12.5 | 6.0 | 2.9 |
| 2024 | OECD (v2) | |  |  | 12.6 | 6.0 | 2.9 |
| 2025 | OECD (v2) | |  |  | 12.6 | 6.0 | 2.9 |
| 2026 | OECD (v2) | |  |  | 12.7 | 5.9 | 2.9 |
| 2027 | OECD (v2) | |  |  | 12.7 | 5.9 | 2.9 |
| 2028 | OECD (v2) | |  |  | 12.7 | 5.9 | 2.9 |
| 2029 | OECD (v2) | |  |  | 12.8 | 5.9 | 2.9 |
| 2030 | OECD (v2) | |  |  | 12.8 | 5.9 | 2.9 |
| 1989 | ADL(≥1) | 9.0 | 5.5 | 3.0 | 6.0 | 3.9 | 1.6 |
| 1990 | ADL(≥1) | 5.6 | 5.8 | 1.3 | 6.1 | 3.9 | 1.6 |
| 1991 | ADL(≥1) | 7.7 | 6.1 | 1.4 | 6.2 | 3.9 | 1.6 |
| 1992 | ADL(≥1) | 5.4 | 3.7 | 1.7 | 6.2 | 4.0 | 1.6 |
| 1993 | ADL(≥1) | 6.1 | 3.6 | 1.2 | 6.3 | 4.0 | 1.7 |
| 1994 | ADL(≥1) | 7.5 | 4.7 | 0.9 | 6.4 | 4.1 | 1.7 |
| 1995 | ADL(≥1) | 7.4 | 4.0 | 0.9 | 6.5 | 4.1 | 1.7 |
| 1996 | ADL(≥1) | 7.1 | 5.1 | 3.3 | 6.6 | 4.2 | 1.7 |
| 1997 | ADL(≥1) | 7.4 | 3.9 | 5.1 | 6.7 | 4.2 | 1.7 |
| 1998 | ADL(≥1) | 4.0 | 4.7 | 0.4 | 6.8 | 4.3 | 1.8 |
| 1999 | ADL(≥1) | 7.2 | 3.9 | 1.9 | 6.9 | 4.3 | 1.8 |
| 2000 | ADL(≥1) | 5.2 | 3.6 | 1.0 | 7.0 | 4.4 | 1.8 |
| 2001 | ADL(≥1) | 7.8 | 4.5 | 1.1 | 7.1 | 4.4 | 1.8 |
| 2002 | ADL(≥1) | 7.9 | 5.8 | 0.4 | 7.2 | 4.5 | 1.8 |
| 2003 | ADL(≥1) | 6.6 | 5.4 | 3.1 | 7.3 | 4.5 | 1.9 |
| 2004 | ADL(≥1) | 9.7 | 4.5 | 1.9 | 7.4 | 4.6 | 1.9 |
| 2005 | ADL(≥1) | 9.0 | 2.3 | 2.2 | 7.6 | 4.6 | 1.9 |
| 2006 | ADL(≥1) | 6.7 | 1.4 | 2.2 | 7.7 | 4.7 | 1.9 |
| 2007 | ADL(≥1) | 6.0 | 5.7 | 1.5 | 7.8 | 4.8 | 1.9 |
| 2008 | ADL(≥1) | 7.3 | 3.3 | 1.6 | 7.9 | 4.8 | 2.0 |
| 2009 | ADL(≥1) | 7.8 | 3.5 | 1.9 | 8.0 | 4.9 | 2.0 |
| 2010 | ADL(≥1) | 7.1 | 3.7 | 3.6 | 8.1 | 4.9 | 2.0 |
| 2011 | ADL(≥1) | 8.3 | 4.3 | 3.3 | 8.2 | 5.0 | 2.0 |
| 2012 | ADL(≥1) | 4.4 | 4.7 | 2.4 | 8.4 | 5.0 | 2.0 |
| 2013 | ADL(≥1) | 6.0 | 4.7 | 0.8 | 8.5 | 5.1 | 2.1 |
| 2014 | ADL(≥1) | 8.2 | 7.2 | 2.2 | 8.6 | 5.2 | 2.1 |
| 2015 | ADL(≥1) | 10.8 | 6.8 | 1.0 | 8.7 | 5.2 | 2.1 |
| 2016 | ADL(≥1) | 11.2 | 5.5 | 3.5 | 8.9 | 5.3 | 2.1 |
| 2017 | ADL(≥1) | 9.3 | 5.5 | 2.3 | 9.0 | 5.3 | 2.2 |
| 2018 | ADL(≥1) | 11.0 | 5.3 | 1.5 | 9.1 | 5.4 | 2.2 |
| 2019 | ADL(≥1) |  |  |  | 9.2 | 5.5 | 2.2 |
| 2020 | ADL(≥1) |  |  |  | 9.4 | 5.5 | 2.2 |
| 2021 | ADL(≥1) |  |  |  | 9.5 | 5.6 | 2.3 |
| 2022 | ADL(≥1) |  |  |  | 9.6 | 5.7 | 2.3 |
| 2023 | ADL(≥1) |  |  |  | 9.8 | 5.7 | 2.3 |
| 2024 | ADL(≥1) |  |  |  | 9.9 | 5.8 | 2.3 |
| 2025 | ADL(≥1) |  |  |  | 10.1 | 5.9 | 2.4 |
| 2026 | ADL(≥1) |  |  |  | 10.2 | 5.9 | 2.4 |
| 2027 | ADL(≥1) |  |  |  | 10.4 | 6.0 | 2.4 |
| 2028 | ADL(≥1) |  |  |  | 10.5 | 6.1 | 2.4 |
| 2029 | ADL(≥1) |  |  |  | 10.6 | 6.1 | 2.5 |
| 2030 | ADL(≥1) |  |  |  | 10.8 | 6.2 | 2.5 |
| Females | | | | | | | |
| 1989 | SRH LG | 34.2 | 29.1 | 28.1 | 35.0 | 27.5 | 22.3 |
| 1990 | SRH LG | 34.9 | 34.6 | 15.9 | 35.2 | 27.6 | 22.3 |
| 1991 | SRH LG | 37.8 | 24.1 | 34.8 | 35.3 | 27.7 | 22.2 |
| 1992 | SRH LG | 34.4 | 22.3 | 11.9 | 35.4 | 27.8 | 22.1 |
| 1993 | SRH LG | 35.3 | 27.1 | 19.7 | 35.6 | 27.8 | 22.1 |
| 1994 | SRH LG | 34.2 | 29.2 | 20.9 | 35.7 | 27.9 | 22.0 |
| 1995 | SRH LG | 35.2 | 29.7 | 17.1 | 35.9 | 28.0 | 22.0 |
| 1996 | SRH LG | 34.4 | 32.3 | 16.9 | 36.0 | 28.1 | 21.9 |
| 1997 | SRH LG | 37.8 | 31.1 | 15.0 | 36.1 | 28.1 | 21.8 |
| 1998 | SRH LG | 35.7 | 27.8 | 26.0 | 36.3 | 28.2 | 21.8 |
| 1999 | SRH LG | 38.6 | 31.1 | 20.2 | 36.4 | 28.3 | 21.7 |
| 2000 | SRH LG | 35.1 | 32.0 | 21.6 | 36.5 | 28.4 | 21.7 |
| 2001 | SRH LG | 33.2 | 25.4 | 29.7 | 36.7 | 28.4 | 21.6 |
| 2002 | SRH LG | 36.1 | 29.1 | 19.7 | 36.8 | 28.5 | 21.6 |
| 2003 | SRH LG | 37.6 | 27.8 | 27.4 | 37.0 | 28.6 | 21.5 |
| 2004 | SRH LG | 37.8 | 29.6 | 22.0 | 37.1 | 28.7 | 21.4 |
| 2005 | SRH LG | 38.0 | 26.0 | 19.0 | 37.2 | 28.7 | 21.4 |
| 2006 | SRH LG | 36.0 | 27.9 | 19.5 | 37.4 | 28.8 | 21.3 |
| 2007 | SRH LG | 36.3 | 24.7 | 19.6 | 37.5 | 28.9 | 21.3 |
| 2008 | SRH LG | 35.6 | 22.9 | 17.3 | 37.7 | 29.0 | 21.2 |
| 2009 | SRH LG | 39.7 | 29.8 | 21.2 | 37.8 | 29.0 | 21.1 |
| 2010 | SRH LG | 38.3 | 26.5 | 20.3 | 37.9 | 29.1 | 21.1 |
| 2011 | SRH LG | 35.6 | 26.6 | 24.6 | 38.1 | 29.2 | 21.0 |
| 2012 | SRH LG | 36.2 | 31.4 | 24.6 | 38.2 | 29.3 | 21.0 |
| 2013 | SRH LG | 37.2 | 27.8 | 18.4 | 38.4 | 29.4 | 20.9 |
| 2014 | SRH LG | 36.8 | 31.7 | 20.2 | 38.5 | 29.4 | 20.9 |
| 2015 | SRH LG | 42.2 | 27.8 | 21.0 | 38.6 | 29.5 | 20.8 |
| 2016 | SRH LG | 40.3 | 30.6 | 19.2 | 38.8 | 29.6 | 20.7 |
| 2017 | SRH LG | 41.1 | 32.2 | 23.4 | 38.9 | 29.7 | 20.7 |
| 2018 | SRH LG | 44.2 | 28.9 | 18.6 | 39.1 | 29.7 | 20.6 |
| 2019 | SRH LG |  |  |  | 39.2 | 29.8 | 20.6 |
| 2020 | SRH LG |  |  |  | 39.3 | 29.9 | 20.5 |
| 2021 | SRH LG |  |  |  | 39.5 | 30.0 | 20.5 |
| 2022 | SRH LG |  |  |  | 39.6 | 30.1 | 20.4 |
| 2023 | SRH LG |  |  |  | 39.8 | 30.1 | 20.3 |
| 2024 | SRH LG |  |  |  | 39.9 | 30.2 | 20.3 |
| 2025 | SRH LG |  |  |  | 40.1 | 30.3 | 20.2 |
| 2026 | SRH LG |  |  |  | 40.2 | 30.4 | 20.2 |
| 2027 | SRH LG |  |  |  | 40.3 | 30.4 | 20.1 |
| 2028 | SRH LG |  |  |  | 40.5 | 30.5 | 20.1 |
| 2029 | SRH LG |  |  |  | 40.6 | 30.6 | 20.0 |
| 2030 | SRH LG |  |  |  | 40.8 | 30.7 | 19.9 |
| 1989 | OECD (≥1) | 26.2 | 19.6 | 13.3 | 25.6 | 18.5 | 13.3 |
| 1990 | OECD (≥1) | 24.9 | 24.7 | 9.2 | 25.6 | 18.4 | 13.2 |
| 1991 | OECD (≥1) | 28.8 | 23.9 | 11.1 | 25.6 | 18.3 | 13.0 |
| 1992 | OECD (≥1) | 27.7 | 12.2 | 11.4 | 25.5 | 18.2 | 12.8 |
| 1993 | OECD (≥1) | 29.7 | 13.3 | 13.4 | 25.5 | 18.1 | 12.7 |
| 1994 | OECD (≥1) | 26.0 | 21.3 | 13.2 | 25.5 | 17.9 | 12.5 |
| 1995 | OECD (≥1) | 28.5 | 19.6 | 17.1 | 25.5 | 17.8 | 12.3 |
| 1996 | OECD (≥1) | 23.1 | 23.1 | 7.0 | 25.5 | 17.7 | 12.2 |
| 1997 | OECD (≥1) | 23.3 | 18.7 | 8.7 | 25.5 | 17.6 | 12.0 |
| 1998 | OECD (≥1) | 22.1 | 18.0 | 13.4 | 25.5 | 17.5 | 11.8 |
| 1999 | OECD (≥1) | 26.1 | 15.0 | 12.3 | 25.5 | 17.3 | 11.7 |
| 2000 | OECD (≥1) | 23.2 | 19.2 | 8.9 | 25.4 | 17.2 | 11.5 |
| 2001 | OECD (≥1) | 21.4 | 13.4 | 15.5 | 25.4 | 17.1 | 11.4 |
| 2002 | OECD (≥1) | 24.2 | 11.9 | 8.0 | 25.4 | 17.0 | 11.2 |
| 2003 | OECD (≥1) | 24.9 | 15.6 | 11.6 | 25.4 | 16.9 | 11.1 |
| 2004 | OECD (≥1) | 24.4 | 19.8 | 11.0 | 25.4 | 16.8 | 10.9 |
| 2005 | OECD (≥1) | 26.5 | 18.7 | 9.3 | 25.4 | 16.7 | 10.8 |
| 2006 | OECD (≥1) | 23.6 | 15.7 | 13.0 | 25.4 | 16.5 | 10.6 |
| 2007 | OECD (≥1) | 24.1 | 14.4 | 13.0 | 25.4 | 16.4 | 10.5 |
| 2008 | OECD (≥1) | 24.8 | 12.6 | 8.9 | 25.3 | 16.3 | 10.4 |
| 2009 | OECD (≥1) | 27.1 | 13.7 | 14.6 | 25.3 | 16.2 | 10.2 |
| 2010 | OECD (≥1) | 27.9 | 13.7 | 12.4 | 25.3 | 16.1 | 10.1 |
| 2011 | OECD (≥1) | 25.2 | 17.2 | 9.4 | 25.3 | 16.0 | 9.9 |
| 2012 | OECD (≥1) | 22.8 | 14.5 | 11.3 | 25.3 | 15.9 | 9.8 |
| 2013 | OECD (≥1) | 28.5 | 18.7 | 8.2 | 25.3 | 15.8 | 9.7 |
| 2014 | OECD (≥1) | 27.2 | 15.2 | 12.7 | 25.3 | 15.7 | 9.6 |
| 2015 | OECD (≥1) | 29.4 | 17.2 | 8.3 | 25.2 | 15.5 | 9.4 |
| 2016 | OECD (≥1) | 26.0 | 15.7 | 7.4 | 25.2 | 15.4 | 9.3 |
| 2017 | OECD (≥1) | 25.6 | 14.2 | 7.7 | 25.2 | 15.3 | 9.2 |
| 2018 | OECD (≥1) | 27.7 | 16.8 | 7.9 | 25.2 | 15.2 | 9.0 |
| 2019 | OECD (≥1) |  |  |  | 25.2 | 15.1 | 8.9 |
| 2020 | OECD (≥1) |  |  |  | 25.2 | 15.0 | 8.8 |
| 2021 | OECD (≥1) |  |  |  | 25.2 | 14.9 | 8.7 |
| 2022 | OECD (≥1) |  |  |  | 25.2 | 14.8 | 8.6 |
| 2023 | OECD (≥1) |  |  |  | 25.1 | 14.7 | 8.4 |
| 2024 | OECD (≥1) |  |  |  | 25.1 | 14.6 | 8.3 |
| 2025 | OECD (≥1) |  |  |  | 25.1 | 14.5 | 8.2 |
| 2026 | OECD (≥1) |  |  |  | 25.1 | 14.4 | 8.1 |
| 2027 | OECD (≥1) |  |  |  | 25.1 | 14.3 | 8.0 |
| 2028 | OECD (≥1) |  |  |  | 25.1 | 14.2 | 7.9 |
| 2029 | OECD (≥1) |  |  |  | 25.1 | 14.1 | 7.8 |
| 2030 | OECD (≥1) |  |  |  | 25.1 | 14.0 | 7.7 |
| 1989 | OECD (v2) | 19.2 | 13.4 | 6.5 | 16.5 | 12.5 | 7.7 |
| 1990 | OECD (v2) | 16.8 | 22.7 | 5.7 | 16.6 | 12.5 | 7.6 |
| 1991 | OECD (v2) | 20.2 | 16.4 | 8.0 | 16.7 | 12.4 | 7.5 |
| 1992 | OECD (v2) | 19.4 | 8.9 | 6.7 | 16.8 | 12.4 | 7.5 |
| 1993 | OECD (v2) | 18.7 | 10.0 | 7.2 | 16.8 | 12.3 | 7.4 |
| 1994 | OECD (v2) | 17.6 | 14.0 | 10.6 | 16.9 | 12.3 | 7.3 |
| 1995 | OECD (v2) | 18.5 | 14.8 | 13.0 | 17.0 | 12.3 | 7.3 |
| 1996 | OECD (v2) | 15.7 | 15.4 | 4.7 | 17.1 | 12.2 | 7.2 |
| 1997 | OECD (v2) | 16.0 | 15.4 | 2.2 | 17.2 | 12.2 | 7.2 |
| 1998 | OECD (v2) | 16.4 | 12.5 | 11.0 | 17.2 | 12.1 | 7.1 |
| 1999 | OECD (v2) | 15.1 | 10.7 | 8.7 | 17.3 | 12.1 | 7.0 |
| 2000 | OECD (v2) | 15.1 | 11.6 | 6.0 | 17.4 | 12.1 | 7.0 |
| 2001 | OECD (v2) | 14.2 | 10.2 | 11.5 | 17.5 | 12.0 | 6.9 |
| 2002 | OECD (v2) | 15.4 | 8.8 | 4.8 | 17.6 | 12.0 | 6.9 |
| 2003 | OECD (v2) | 16.8 | 13.1 | 6.6 | 17.6 | 12.0 | 6.8 |
| 2004 | OECD (v2) | 17.2 | 13.9 | 4.7 | 17.7 | 11.9 | 6.8 |
| 2005 | OECD (v2) | 17.5 | 10.7 | 6.2 | 17.8 | 11.9 | 6.7 |
| 2006 | OECD (v2) | 16.2 | 9.2 | 6.9 | 17.9 | 11.8 | 6.7 |
| 2007 | OECD (v2) | 15.4 | 8.0 | 7.4 | 18.0 | 11.8 | 6.6 |
| 2008 | OECD (v2) | 16.2 | 7.6 | 4.6 | 18.0 | 11.8 | 6.5 |
| 2009 | OECD (v2) | 19.6 | 9.5 | 5.9 | 18.1 | 11.7 | 6.5 |
| 2010 | OECD (v2) | 17.2 | 6.0 | 4.4 | 18.2 | 11.7 | 6.4 |
| 2011 | OECD (v2) | 13.6 | 7.2 | 3.8 | 18.3 | 11.6 | 6.4 |
| 2012 | OECD (v2) | 15.5 | 9.9 | 5.3 | 18.4 | 11.6 | 6.3 |
| 2013 | OECD (v2) | 15.7 | 9.5 | 2.7 | 18.4 | 11.6 | 6.3 |
| 2014 | OECD (v2) | 22.2 | 12.2 | 8.0 | 18.5 | 11.5 | 6.2 |
| 2015 | OECD (v2) | 23.2 | 12.0 | 6.5 | 18.6 | 11.5 | 6.2 |
| 2016 | OECD (v2) | 21.6 | 13.2 | 6.7 | 18.7 | 11.5 | 6.1 |
| 2017 | OECD (v2) | 22.0 | 11.4 | 5.8 | 18.8 | 11.4 | 6.1 |
| 2018 | OECD (v2) | 22.2 | 14.1 | 6.5 | 18.9 | 11.4 | 6.0 |
| 2019 | OECD (v2) | |  |  | 19.0 | 11.4 | 6.0 |
| 2020 | OECD (v2) | |  |  | 19.0 | 11.3 | 5.9 |
| 2021 | OECD (v2) | |  |  | 19.1 | 11.3 | 5.9 |
| 2022 | OECD (v2) | |  |  | 19.2 | 11.2 | 5.8 |
| 2023 | OECD (v2) | |  |  | 19.3 | 11.2 | 5.8 |
| 2024 | OECD (v2) | |  |  | 19.4 | 11.2 | 5.7 |
| 2025 | OECD (v2) | |  |  | 19.5 | 11.1 | 5.7 |
| 2026 | OECD (v2) | |  |  | 19.6 | 11.1 | 5.6 |
| 2027 | OECD (v2) | |  |  | 19.6 | 11.1 | 5.6 |
| 2028 | OECD (v2) | |  |  | 19.7 | 11.0 | 5.5 |
| 2029 | OECD (v2) | |  |  | 19.8 | 11.0 | 5.5 |
| 2030 | OECD (v2) | |  |  | 19.9 | 11.0 | 5.4 |
| 1989 | ADL(≥1) | 6.3 | 4.5 | 0.0 | 8.1 | 5.9 | 5.1 |
| 1990 | ADL(≥1) | 6.1 | 12.9 | 5.9 | 8.2 | 6.0 | 5.0 |
| 1991 | ADL(≥1) | 10.2 | 8.4 | 5.1 | 8.3 | 6.0 | 5.0 |
| 1992 | ADL(≥1) | 7.3 | 5.0 | 2.8 | 8.3 | 6.0 | 4.9 |
| 1993 | ADL(≥1) | 8.5 | 3.3 | 6.6 | 8.4 | 6.1 | 4.9 |
| 1994 | ADL(≥1) | 8.0 | 10.3 | 6.4 | 8.5 | 6.1 | 4.8 |
| 1995 | ADL(≥1) | 10.2 | 4.6 | 8.0 | 8.6 | 6.1 | 4.7 |
| 1996 | ADL(≥1) | 8.6 | 4.4 | 1.9 | 8.7 | 6.2 | 4.7 |
| 1997 | ADL(≥1) | 10.2 | 10.1 | 1.1 | 8.8 | 6.2 | 4.6 |
| 1998 | ADL(≥1) | 9.0 | 8.1 | 2.6 | 8.9 | 6.2 | 4.6 |
| 1999 | ADL(≥1) | 7.0 | 3.3 | 3.1 | 9.0 | 6.3 | 4.5 |
| 2000 | ADL(≥1) | 9.2 | 6.8 | 1.7 | 9.1 | 6.3 | 4.4 |
| 2001 | ADL(≥1) | 8.2 | 5.0 | 5.9 | 9.2 | 6.3 | 4.4 |
| 2002 | ADL(≥1) | 7.9 | 6.0 | 8.3 | 9.3 | 6.4 | 4.3 |
| 2003 | ADL(≥1) | 9.1 | 8.3 | 2.5 | 9.4 | 6.4 | 4.3 |
| 2004 | ADL(≥1) | 11.0 | 6.9 | 4.2 | 9.5 | 6.4 | 4.2 |
| 2005 | ADL(≥1) | 9.1 | 3.9 | 5.0 | 9.6 | 6.5 | 4.2 |
| 2006 | ADL(≥1) | 11.5 | 4.2 | 8.5 | 9.7 | 6.5 | 4.1 |
| 2007 | ADL(≥1) | 11.6 | 3.6 | 5.2 | 9.8 | 6.5 | 4.1 |
| 2008 | ADL(≥1) | 8.0 | 4.6 | 2.4 | 9.9 | 6.6 | 4.0 |
| 2009 | ADL(≥1) | 8.8 | 3.1 | 2.9 | 10.0 | 6.6 | 4.0 |
| 2010 | ADL(≥1) | 8.8 | 4.4 | 3.1 | 10.1 | 6.7 | 3.9 |
| 2011 | ADL(≥1) | 7.2 | 4.0 | 1.4 | 10.3 | 6.7 | 3.9 |
| 2012 | ADL(≥1) | 4.8 | 4.0 | 4.9 | 10.4 | 6.7 | 3.8 |
| 2013 | ADL(≥1) | 8.9 | 6.4 | 3.4 | 10.5 | 6.8 | 3.8 |
| 2014 | ADL(≥1) | 11.2 | 7.8 | 5.1 | 10.6 | 6.8 | 3.7 |
| 2015 | ADL(≥1) | 12.8 | 7.8 | 3.1 | 10.7 | 6.8 | 3.7 |
| 2016 | ADL(≥1) | 10.2 | 8.6 | 3.0 | 10.8 | 6.9 | 3.6 |
| 2017 | ADL(≥1) | 12.8 | 6.9 | 2.3 | 10.9 | 6.9 | 3.6 |
| 2018 | ADL(≥1) | 13.0 | 7.2 | 4.2 | 11.0 | 7.0 | 3.5 |
| 2019 | ADL(≥1) |  |  |  | 11.2 | 7.0 | 3.5 |
| 2020 | ADL(≥1) |  |  |  | 11.3 | 7.0 | 3.4 |
| 2021 | ADL(≥1) |  |  |  | 11.4 | 7.1 | 3.4 |
| 2022 | ADL(≥1) |  |  |  | 11.5 | 7.1 | 3.4 |
| 2023 | ADL(≥1) |  |  |  | 11.6 | 7.2 | 3.3 |
| 2024 | ADL(≥1) |  |  |  | 11.8 | 7.2 | 3.3 |
| 2025 | ADL(≥1) |  |  |  | 11.9 | 7.2 | 3.2 |
| 2026 | ADL(≥1) |  |  |  | 12.0 | 7.3 | 3.2 |
| 2027 | ADL(≥1) |  |  |  | 12.1 | 7.3 | 3.2 |
| 2028 | ADL(≥1) |  |  |  | 12.3 | 7.4 | 3.1 |
| 2029 | ADL(≥1) |  |  |  | 12.4 | 7.4 | 3.1 |
| 2030 | ADL(≥1) |  |  |  | 12.5 | 7.4 | 3.0 |

^a T^he age standardized prevalence uses the European Union Standard Population for 2013, and data from the Dutch Health survey (1989-2018). ^b^ Model predicted prevalences by logistic models including age category (50-54;..;65-69), education level (low, medium, high), year of the survey, and interaction term between education and year. Health indicators included are: less than good SAH(SAH), Organization for Economic Cooperation and Development (OECD) functional limitation indicator, OECD indicator without hearing and seeing items, and Activities of Daily living (ADLs)

**Appendix Table 6. Years in good health for several health indicators for individuals aged 50-69, by gender, education and year**

| **Men** |  | **Partial life expectancy** | | | **Healthy years** | | |
| --- | --- | --- | --- | --- | --- | --- | --- |
| **Education** | **Indicator** | **2006** | **2018** | **2030** | **2006** | **2018** | **2030** |
| Low | Less than good SAH | 18.31 | 18.55 | 18.66 | 11.21 | 11.23 | 11.18 |
| Low | OECD | 18.31 | 18.55 | 18.66 | 14.33 | 14.74 | 15.05 |
| Low | OECD w-o hear see | 18.31 | 18.55 | 18.66 | 16.14 | 16.25 | 16.26 |
| Low | ADL | 18.31 | 18.55 | 18.66 | 17.28 | 17.30 | 17.18 |
| Med | Less than good SAH | 18.69 | 19.01 | 19.23 | 13.76 | 14.20 | 14.58 |
| Med | OECD | 18.69 | 19.01 | 19.23 | 16.27 | 16.88 | 17.38 |
| Med | OECD w-o hear see | 18.69 | 19.01 | 19.23 | 17.50 | 17.84 | 18.10 |
| Med | ADL | 18.69 | 19.01 | 19.23 | 18.04 | 18.24 | 18.34 |
| High | Less than good SAH | 19.14 | 19.31 | 19.44 | 15.76 | 15.88 | 15.97 |
| High | OECD | 19.14 | 19.31 | 19.44 | 17.66 | 18.11 | 18.47 |
| High | OECD w-o hear see | 19.14 | 19.31 | 19.44 | 18.61 | 18.75 | 18.87 |
| High | ADL | 19.14 | 19.31 | 19.44 | 18.87 | 18.99 | 19.08 |
| **Women** | **Indicator** | **Partial life expectancy** | | | **Healthy years** | | |
| Low | Less than good SAH | 18.96 | 18.92 | 18.88 | 11.83 | 11.49 | 11.14 |
| Low | OECD | 18.96 | 18.92 | 18.88 | 14.13 | 14.13 | 14.13 |
| Low | OECD w-o hear see | 18.96 | 18.92 | 18.88 | 15.53 | 15.31 | 15.08 |
| Low | ADL | 18.96 | 18.92 | 18.88 | 17.59 | 17.36 | 17.12 |
| Med | Less than good SAH | 19.20 | 19.31 | 19.42 | 13.63 | 13.52 | 13.42 |
| Med | OECD | 19.20 | 19.31 | 19.42 | 16.01 | 16.35 | 16.68 |
| Med | OECD w-o hear see | 19.20 | 19.31 | 19.42 | 16.90 | 17.08 | 17.26 |
| Med | ADL | 19.20 | 19.31 | 19.42 | 18.27 | 18.30 | 18.33 |
| High | Less than good SAH | 19.40 | 19.49 | 19.56 | 15.23 | 15.43 | 15.62 |
| High | OECD | 19.40 | 19.49 | 19.56 | 17.32 | 17.71 | 18.05 |
| High | OECD w-o hear see | 19.40 | 19.49 | 19.56 | 18.08 | 18.29 | 18.48 |
| High | ADL | 19.40 | 19.49 | 19.56 | 18.79 | 18.97 | 19.11 |

Years in good health obtained from combining the predicted prevalences by logistic models from the Dutch Health Interview Survey and projected mortality rates

The logistic models included age category (50-54;..;65-69), education level (low, medium, high), year of the survey, and interaction term between education and year. Health indicators included are: less than good SAH(SAH), Organization for Economic Cooperation and Development (OECD) functional limitation indicator, OECD indicator without hearing and seeing items, and Activities of Daily living (ADLs)


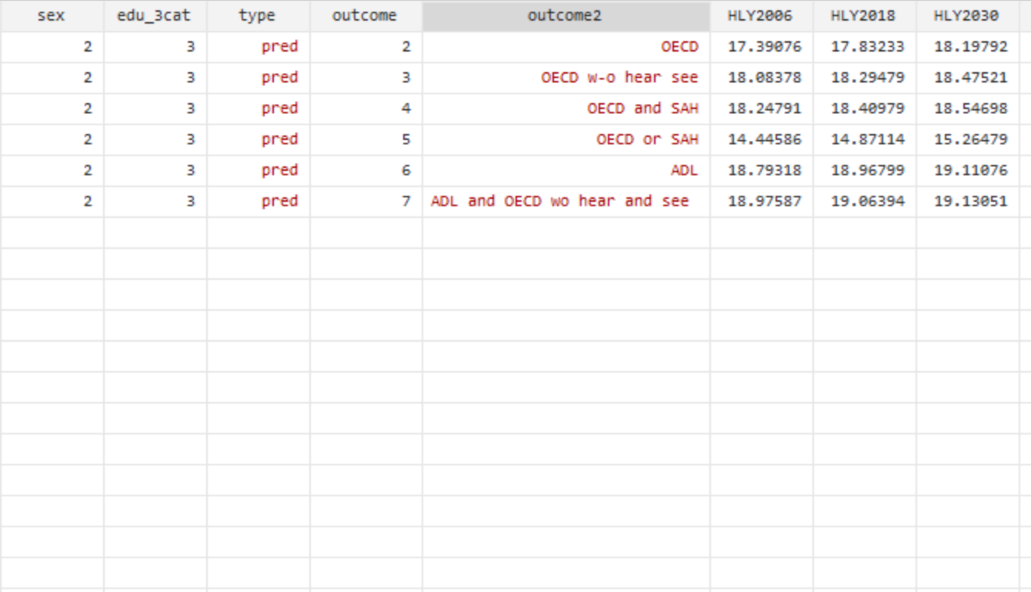


**Appendix Table 7. Surplus or deficit of years in good health relative to the statutory retirement age in the Netherlands and educational inequalities , by gender, education and year**

| **Men** |  | **Deficit/Surplus of healthy years** | | | **Inequality in Deficit/Surplus (low-high)** | | |
| --- | --- | --- | --- | --- | --- | --- | --- |
| **Education** | **Indicator** | **2006** | **2018** | **2030** | **2006** | **2018** | **2030** |
| Low | Less than good SAH | -3.8 | -4.8 | -6.1 | 4.5 | 4.6 | 4.8 |
| Low | OECD | -0.7 | -1.3 | -2.2 | 3.3 | 3.4 | 3.4 |
| Low | OECD w-o hear see | 1.1 | 0.3 | -1.0 | 2.5 | 2.5 | 2.6 |
| Low | ADL | 2.3 | 1.3 | -0.1 | 1.6 | 1.7 | 1.9 |
| Med | Less than good SAH | -1.2 | -1.8 | -2.7 | 4.5 | 4.6 | 4.8 |
| Med | OECD | 1.3 | 0.9 | 0.1 | 3.3 | 3.4 | 3.4 |
| Med | OECD w-o hear see | 2.5 | 1.8 | 0.8 | 2.5 | 2.5 | 2.6 |
| Med | ADL | 3.0 | 2.2 | 1.1 | 1.6 | 1.7 | 1.9 |
| High | Less than good SAH | 0.8 | -0.1 | -1.3 | 4.5 | 4.6 | 4.8 |
| High | OECD | 2.7 | 2.1 | 1.2 | 3.3 | 3.4 | 3.4 |
| High | OECD w-o hear see | 3.6 | 2.8 | 1.6 | 2.5 | 2.5 | 2.6 |
| High | ADL | 3.9 | 3.0 | 1.8 | 1.6 | 1.7 | 1.9 |
| **Women** |  |  |  |  |  |  |  |
| Low | Less than good SAH | -3.2 | -4.5 | -6.1 | 3.4 | 3.9 | 4.5 |
| Low | OECD | -0.9 | -1.9 | -3.1 | 3.2 | 3.6 | 3.9 |
| Low | OECD w-o hear see | 0.5 | -0.7 | -2.2 | 2.6 | 3.0 | 3.4 |
| Low | ADL | 2.6 | 1.4 | -0.1 | 1.2 | 1.6 | 2.0 |
| Med | Less than good SAH | -1.4 | -2.5 | -3.8 | 3.4 | 3.9 | 4.5 |
| Med | OECD | 1.0 | 0.3 | -0.6 | 3.2 | 3.6 | 3.9 |
| Med | OECD w-o hear see | 1.9 | 1.1 | 0.0 | 2.6 | 3.0 | 3.4 |
| Med | ADL | 3.3 | 2.3 | 1.1 | 1.2 | 1.6 | 2.0 |
| High | Less than good SAH | 0.2 | -0.6 | -1.6 | 3.4 | 3.9 | 4.5 |
| High | OECD | 2.3 | 1.7 | 0.8 | 3.2 | 3.6 | 3.9 |
| High | OECD w-o hear see | 3.1 | 2.3 | 1.2 | 2.6 | 3.0 | 3.4 |
| High | ADL | 3.8 | 3.0 | 1.9 | 1.2 | 1.6 | 2.0 |

The difference between years in good health for each health indicator and the years remaining to the retirement age from age 50 at these three points in time: (Years in good health between ages 50 and 70– (retirement age-50)). In 2006 the retirement age was 65 years, in 2018 it was 66 years and in 2030 it will be 67 years and 3 months. We present the `deficit’ of years in good health to reach the retirement age in good health or `surplus’ of years in good health expected to live past the retirement age. Health indicators included are: less than good SAH(SAH), Organization for Economic Cooperation and Development (OECD) functional limitation indicator, OECD indicator without hearing and seeing items, and Activities of Daily living (ADLs)

**Appendix 8. Partial Life expectancy, healthy life years and `deficit’ or `surplus’ for the medium educated between ages 50-69 by gender.**

Men – Medium Educated Women – Medium Educated

Men – Medium Educated Women – Medium Educated

The difference between years in good health for each health indicator and the years remaining to the retirement age from age 50 at these three points in time: (Years in good health between ages 50 and 70– (retirement age-50)). In 2006 the retirement age was 65 years, in 2018 it was 66 years and in 2030 it will be 67 years and 3 months. We present the `deficit’ of years in good health to reach the retirement age in good health or `surplus’ of years in good health expected to live past the retirement age. Health indicators included are: less than good SAH(SAH), Organization for Economic Cooperation and Development (OECD) functional limitation indicator, OECD indicator without hearing and seeing items, and Activities of Daily living (ADLs)

**Appendix Table 9 - Robustness - `Deficit’ and `Surplus’ of years in good health relative to the retirement age for different health indicators for individuals between 50-69 by year, gender, education and related educational inequalities – Alternative scenarios**

**Low Educated Men**

Main Results

Constant disability

Constant mortality

Constant mortality and disability

**Low Educated Women**

Constant mortality and disability

Constant mortality

Constant disability

Main Results

**Robustness - `Deficit’ and `Surplus’ of years in good health relative to the retirement age for different health indicators for individuals between 50-69 by year, gender, education and related educational inequalities – Alternative scenarios**

**High Educated Men**

**Appendix Table 9 - Robustness - `Deficit’ and `Surplus’ of years in good health relative to the retirement age for different health indicators for individuals between 50-69 by year, gender, education and related educational inequalities – Alternative scenarios**

**High Educated Men**

S3. Constant disability

S1.Constant mortality and disability

S2. Constant mortality

Main Results

**High Educated Women**

S3. Constant disability

S2. Constant mortality

S1.Constant mortality and disability

Main Results

**Robustness – Educational inequalities in `Deficit’ and `Surplus’ of years in good health relative to the retirement age for different health indicators for individuals between 50-69 by year, gender – Alternative scenarios**

| ***Men*** |  |  |  |  |  |  |  |  |  |
| --- | --- | --- | --- | --- | --- | --- | --- | --- | --- |
| SAH | ***Main*** | ***(S1) constant both*** | ***(S2) - constant mortality*** | ***(S3) – constant poor health*** | ***Main – (S1)*** | ***(S2) –***  ***(S1)*** | ***(S3) –***  ***(S1)*** | ***% poor health*** | ***%***  ***mortality*** |
| 2006 | 4.5 | 4.5 | 4.5 | 4.5 | 0 | 0 | 0 |  |  |
| 2018 | 4.6 | 4.5 | 4.6 | 4.5 | 0.1 | 0.1 | 0 | 100 | 0 |
| 2030 | 4.8 | 4.5 | 4.8 | 4.5 | 0.3 | 0.3 | 0 | 100 | 0 |
| OECD |  |  |  |  |  |  |  |  |  |
| 2006 | 3.2 | 3.2 | 3.2 | 3.2 | 0 | 0 | 0 |  |  |
| 2018 | 3.2 | 3.2 | 3.2 | 3.2 | 0 | 0 | 0 | 0 | 0 |
| 2030 | 3.2 | 3.2 | 3.2 | 3.2 | 0 | 0 | 0 | 0 | 0 |
| OECD w/o hearing and seeing | | |  |  |  |  |  |  |  |
| 2006 | 2.5 | 2.5 | 2.5 | 2.5 | 0 | 0 | 0 |  |  |
| 2018 | 2.5 | 2.5 | 2.5 | 2.5 | 0 | 0 | 0 | 0 | 0 |
| 2030 | 2.6 | 2.5 | 2.6 | 2.5 | 0.1 | 0.1 | 0 | 100 | 0 |
| ADL |  |  |  |  |  |  |  |  |  |
| 2006 | 1.6 | 1.6 | 1.6 | 1.6 | 0 | 0 | 0 |  |  |
| 2018 | 1.7 | 1.6 | 1.7 | 1.6 | 0.1 | 0.1 | 0 | 100 | 0 |
| 2030 | 1.9 | 1.6 | 1.9 | 1.6 | 0.3 | 0.3 | 0 | 100 | 0 |

The difference between years in good health for each health indicator and the years remaining to the retirement age from age 50 at these three points in time: (Years in good health between ages 50 and 70– (retirement age-50)). In 2006 the retirement age was 65 years, in 2018 it was 66 years and in 2030 it will be 67 years and 3 months. We present the `deficit’ of years in good health to reach the retirement age in good health or `surplus’ of years in good health expected to live past the retirement age. Health indicators included are: less than good SAH(SAH), Organization for Economic Cooperation and Development (OECD) functional limitation indicator, OECD indicator without hearing and seeing items, and Activities of Daily living (ADLs)

Scenario 1 – assumes mortality and disability are constant at 2006 levels

Scenario 2 – assumes constant mortality at 2006 levels

Scenario 3 – assumes constant poor health at 2006 level

**Robustness - Educational inequalities in `Deficit’ and `Surplus’ of years in good health relative to the retirement age for different health indicators for individuals between 50-69 by year, gender – Alternative scenarios**

| ***Women*** |  |  |  |  |  |  |  |  |  |
| --- | --- | --- | --- | --- | --- | --- | --- | --- | --- |
| SAH | ***Main*** | ***(S1) constant both*** | ***(S2) - constant mortality*** | ***(S3) – constant poor health*** | ***Main – (S1)*** | ***(S2) –***  ***(S1)*** | ***(S3) –***  ***(S1)*** | ***% poor health*** | ***%***  ***mortality*** |
| 2006 | 3.4 | 3.4 | 3.4 | 3.4 | 0 | 0 | 0 |  |  |
| 2018 | 3.9 | 3.4 | 3.8 | 3.5 | 0.5 | 0.4 | 0.1 | 80 | 20 |
| 2030 | 4.5 | 3.4 | 4.3 | 3.6 | 1.1 | 0.9 | 0.2 | 81.8 | 18.2 |
| OECD |  |  |  |  |  |  |  |  |  |
| 2006 | 3.1 | 3.1 | 3.1 | 3.1 | 0 | 0 | 0 |  |  |
| 2018 | 3.5 | 3.1 | 3.4 | 3.2 | 0.4 | 0.3 | 0.1 | 75 | 25 |
| 2030 | 3.9 | 3.1 | 3.7 | 3.3 | 0.8 | 0.6 | 0.2 | 75 | 25 |
| OECD w/o hearing and seeing | | |  |  |  |  |  |  |  |
| 2006 | 2.6 | 2.6 | 2.6 | 2.6 | 0 | 0 | 0 |  |  |
| 2018 | 3 | 2.6 | 2.9 | 2.7 | 0.4 | 0.3 | 0.1 | 75 | 25 |
| 2030 | 3.4 | 2.6 | 3.2 | 2.8 | 0.8 | 0.6 | 0.2 | 75 | 25 |
| ADL |  |  |  |  |  |  |  |  |  |
| 2006 | 1.2 | 1.2 | 1.2 | 1.2 | 0 | 0 | 0 |  |  |
| 2018 | 1.6 | 1.2 | 1.5 | 1.3 | 0.4 | 0.3 | 0.1 | 75 | 25 |
| 2030 | 2 | 1.2 | 1.8 | 1.4 | 0.8 | 0.6 | 0.2 | 75 | 25 |

The difference between years in good health for each health indicator and the years remaining to the retirement age from age 50 at these three points in time: (Years in good health between ages 50 and 70– (retirement age-50)). In 2006 the retirement age was 65 years, in 2018 it was 66 years and in 2030 it will be 67 years and 3 months. We present the `deficit’ of years in good health to reach the retirement age in good health or `surplus’ of years in good health expected to live past the retirement age. Health indicators included are: less than good SAH(SAH), Organization for Economic Cooperation and Development (OECD) functional limitation indicator, OECD indicator without hearing and seeing items, and Activities of Daily living (ADLs)

Scenario 1 – assumes mortality and disability are constant at 2006 levels

Scenario 2 – assumes constant mortality at 2006 levels

Scenario 3 – assumes constant poor health at 2006 levels
